# Gut microbiota–derived metabolite 3-idoleacetic acid together with LPS induces IL-35^+^ B cell generation

**DOI:** 10.1186/s40168-021-01205-8

**Published:** 2022-01-24

**Authors:** Xiaomin Su, Minying Zhang, Houbao Qi, Yunhuan Gao, Yazheng Yang, Huan Yun, Qianjing Zhang, Xiaorong Yang, Yuan Zhang, Jiangshan He, Yaqi Fan, Yuxue Wang, Pei Guo, Chunze Zhang, Rongcun Yang

**Affiliations:** 1 Department of Immunology, Nankai University School of Medicine, Nankai University, Tianjin, 300071 China; 2 Translational Medicine Institute, Affiliated Tianjin Union Medical Center of Nankai University, Tianjin, China; 3 Department of Colorectal Surgery, Tianjin Union Medical Center, Tianjin, 300121 China; 4 State Key Laboratory of Medicinal Chemical Biology, Nankai University, Tianjin, 300071 China

**Keywords:** Reg4, IAA, Gut microbiota, *Lactobacillus*, IL-35^+^ B cells, PXR, TLR4

## Abstract

**Background:**

IL-35–producing Bregs and Treg cells critically regulate chronic illnesses worldwide via mechanisms related to disrupting the gut microbiota composition. However, whether the gut microbiota regulates these IL-35^+^ cells remains elusive. We herein investigated the regulatory effects of the gut microbiota on IL-35^+^ cells by using genetically modified mouse models of obesity.

**Results:**

We first found that gut Reg4 promoted resistance to high-fat diet-induced obesity. Using 16S rRNA sequencing combined with LC-MS (liquid chromatography–mass spectrometry)/MS, we demonstrated that gut Reg4 associated with bacteria such as *Lactobacillus* promoted the generation of IL-35^+^ B cells through 3-idoleacetic acid (IAA) in the presence of LPS. *HuREG4*^*IECtg*^ mice fed a high-fat diet exhibited marked IL-35^+^ cell accumulation in not only their adipose tissues but also their colons, whereas decreased IL-35^+^ cell accumulation was observed in the adipose and colon tissues of *Reg4* knockout (KO) mice. We also found that Reg4 mediated HFD-induced obesity resistance via IL-35. Lower levels of IAA were also detected in the peripheral blood of individuals with obesity compared with nonobese subjects. Mechanistically, IAA together with LPS mediated IL-35^+^ B cells through PXR and TLR4. KO of *PXR* or *TLR4* impaired the generation of IL-35^+^ B cells.

**Conclusion:**

Together, IAA and LPS induce the generation of IL-35^+^ B cells through PXR and TLR4.

Video Abstract

**Supplementary Information:**

The online version contains supplementary material available at 10.1186/s40168-021-01205-8.

## Introduction

The gut microbiota can influence essential human functions, including inflammation, digestion, and energy metabolism, by modulating the immune pathways and neural and endocrine systems of the host [1–4]. Disruption of the microbiota composition and function by factors such as genetics are thought to be critical for the progression of chronic illnesses such as metabolic diseases, which are related to the IL-35–producing Bregs and Treg cells [5–10]. However, how alteration of the microbiota influences the development and outcomes of metabolic diseases is incompletely characterized. Gut microbiota/metabolites can affect the differentiation and development of immune cells. Multiple transcription factors, such as aryl hydrocarbon receptor (AhR) [11, 12], Foxp3, and RORγ [8, 13, 14], are involved in this process; for example, AhR contributes to IL-22 transcription [11] through the AhR ligand indole-3-aldehyde, which is produced by *Lactobacillus reuteri*. Studies in animal models and humans have demonstrated that gastrointestinal bacteria/metabolites also participate in B cell differentiation, maturation, and activation [15, 16]; for example, aryl hydrocarbon contributes to the transcriptional programming of IL-10-producing regulatory B cells [17].

IL-35–producing B regulatory (Breg) cells are critical regulators of immunity in multiple diseases, such as autoimmune and infectious diseases, and of cancer progression [9, 17–20]*.* IL-35, a potent anti-inflammatory cytokine, is a newly identified member of the IL-12 family of heterodimeric cytokines comprised of p35 (IL-12A), which is shared by both IL-35 and IL-12, and Epstein–Barr virus-induced gene 3 (Ebi3), which is shared by IL-27 and IL-35 [21]. This cytokine has strong suppressive properties both *in vivo* and *in vitro* [22–24]. It can exert wide-ranging effects on multiple types of immune cells, such as T cells, B cells, macrophages, and dendritic cells (DCs) [19], promote the generation of Treg cells and anti-inflammatory macrophage 2 (M2) [25, 26], and impede the differentiation of Th1 cells [27]. The expression of IL-35 is dysregulated in inflammatory autoimmune diseases such as systemic lupus erythematosus, rheumatoid arthritis, inflammatory bowel disease, multiple sclerosis, type 1 diabetes, psoriasis, multiple sclerosis, autoimmune hepatitis, and experimental autoimmune uveitis [28]. Some CD4^+^Foxp3^+^ regulatory T cells (Tregs) [29], CD8^+^ Tregs [30], tissue macrophages [26], and DCs [31] can also produce IL-35. However, whether gut microbiota/metabolites regulate the differentiation and generation of IL-35^+^ cells has not been determined.

Gut epithelial cells can produce bactericidal substances such as the regenerating gene (Reg) family, lysozyme 1, lysozyme 2, secretory phospholipase A2, α-defensins (cryptdins), and cryptdin-related proteins, which play a critical role in not only eliminating pathogens but also maintaining gut microbiota homeostasis. Reg4 expression is restricted in Paneth cells at the bottom of crypts and also is observed in enteroendocrine cells in the villus [32, 33]. This protein, which adopts a typical lectin fold and binds mannose with two calcium-independent sites [34], damages the bacterial cell wall [34, 35]. Here, we found that Reg4 expressed in gut epithelial cells affected the gut microbiota composition, especially by increasing the proportion of *Lactobacillus*. The metabolite 3-idoleacetic acid (IAA) produced by the increased proportions of *Lactobacillus* can promote the generation and accumulation of IL-35^+^ B cells in not only adipose tissues but also gut tissues and other organs to maintain immune homeostasis.

## Results

### Reg4 promotes resistance to high-fat diet–induced obesity

**Fig. 1:**
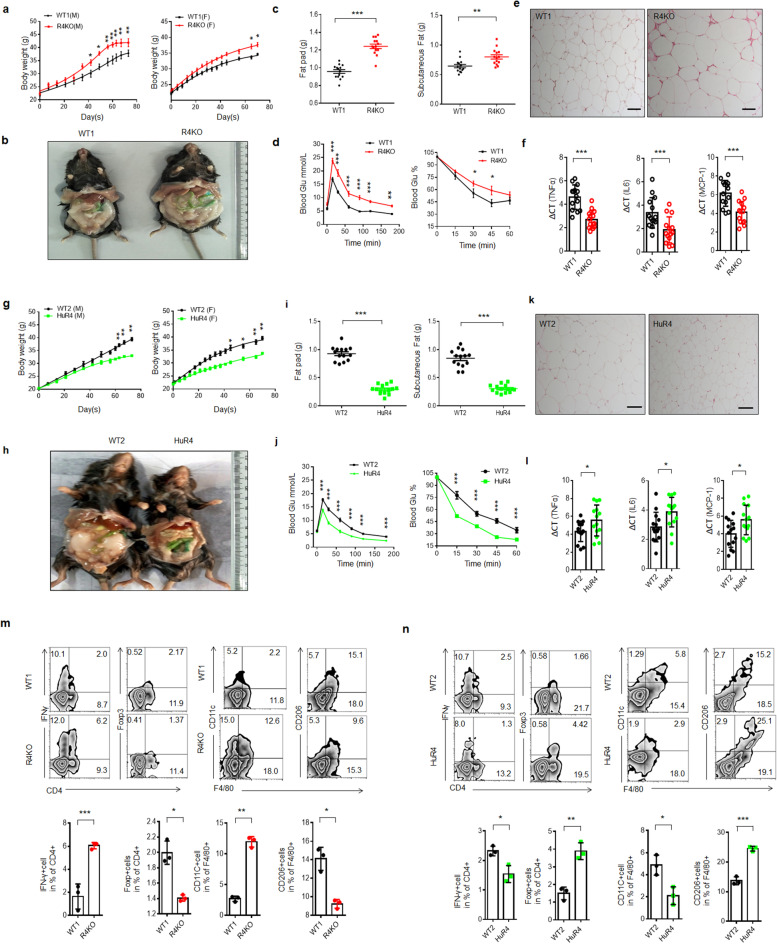
Reg4 promotes resistance to high-fat diet-induced obesity. **a** Body weight increases in male (left) or female (right) WT1 and *Reg4* KO mice (R4KO) fed a high-fat diet (HFD) (*n* = 14). The body weights of these mice did not differ at baseline before HFD feeding. **b** Typical phenotypes of male WT1 and *Reg4* KO mice (R4KO) fed a HFD for 3 months. **c** Fat pad weights of WT1 and *Reg4*KO mice (R4KO) fed a HFD (*n* = 14). **d** Glucose tolerance and insulin sensitivity of WT1 and *Reg4* KO mice (R4KO) fed a HFD for 3 months (*n* = 6). **e** H/E staining of the adipose tissues of WT1 and *Reg4* KO mice (R4KO) fed a HFD. **f** qRT-PCR of TNFα, IL6, and MCP-1 in the adipose tissues of WT1 and *Reg4* KO mice (R4KO) fed a HFD (*n* = 14). **g** Body weight increases in male (left) and female (right) WT2 and *huREG4*^*IECtg*^ mice (HuR4) fed a HFD (*n* = 14). The body weights of these mice did not differ at baseline before HFD feeding. **h** Typical phenotypes of male WT2 and *hu*^*REG4IECtg*^ mice (HuR4) fed a HFD for 3 months. **i** Fat pad weights of WT2 and *huREG4*^*IECtg*^ mice (HuR4) fed a HFD (*n* = 14). **j** Glucose tolerance and insulin sensitivity of WT2 and *huREG4*^*IECtg*^ mice (HuR4) fed a HFD (*n* = 6). **k** H/E staining of the adipose tissues of WT2 and *huREG4*^*IECtg*^ mice (HuR4) fed a HFD. **l** qRT-PCR of TNFα, IL6, and MCP-1 in the adipose tissues of WT2 and *huREG4*^*IECtg*^ mice (HuR4) fed a HFD (*n* = 14). **m** Flow cytometry of IFNγ^+^CD4^+^, Foxp3^+^CD4^+^, F4/80^+^CD11C^+^, and F4/80^+^CD206^+^ cells in the adipose tissues of *Reg4* KO (R4KO) and control WT mice (WT1) fed a HFD for 3 months. **n** Flow cytometry of IFNγ^+^CD4^+^, Foxp3^+^CD4^+^, F4/80^+^CD11C^+^, and F4/80^+^CD206^+^ cells in the adipose tissues of *huREG4*^*IECtg*^ (HuR4) and control littermate WT mice (WT2) fed a HFD for 3 months. The data in **a**–**l** are representative of three independent experiments; the data in **m** and **n** are from three independent experiments. Scale bars = 40 μm; analysis of variance in **a**, **d**, **g**, and **j**; Student’s *t* test in other panels, mean ± SD; *p < 0.05, **p < 0.01, and ***p < 0.001

We previously reported that Reg4 could kill *Escherichia coli* through a complement-dependent pathway [36]. Since alteration of the gut microbiota is related to the occurrence and development of multiple diseases, such as obesity [37–39], we further investigated the role of Reg4 in high-fat diet (HFD)–mediated obesity using *Reg4* knockout (KO) mice. We found that these *Reg4* KO mice showed more sensitivity to HFD-induced obesity, including a higher body weight, higher fat pad tissue weight, decreased insulin sensitivity and reduced glucose tolerance (Fig. 1a–d). Notably, they were not remarkably different from their control wild-type littermates fed normal chow (Fig. S1a,b). Histochemical staining showed larger adipose cells in *Reg4* KO mice than in WT mice (Fig. 1e). The levels of inflammatory cytokines related to the development of obesity [40], such as TNFα, IL-6, and MCP-1, were higher in the adipose tissues of *Reg4* KO mice than in those of the control mice (Fig. 1f). Since Reg4/REG4 is highly homologous between mice and humans (66% amino acid sequence homology), we also generated transgenic mice expressing *REG4* in their intestinal epithelial cells (*huREG4*^*IECtg*^) (Fig. S1c-e) to further investigate the role of gut Reg4 in HFD-mediated obesity. In these mice, the human *REG4* gene was specifically expressed in mouse gut epithelial cells, especially in Paneth cells. These *huREG4*^*IECtg*^ mice were not markedly different from their WT control littermates when fed normal chow (Fig. S1f, g). However, when fed a HFD, the *huREG4*^*IECtg*^ mice showed marked resistance to HFD-induced obesity, including reduced whole body and fat pad tissue weights and increased insulin sensitivity and glucose tolerance (Fig. 1g-j). Histochemical staining showed smaller adipose cells in *huREG4*^*IECtg*^ mice than in WT mice (Fig. 1k). The levels of inflammatory cytokines such as TNFα, IL6 and MCP-1 were also reduced in the adipose tissues of *huREG4*^*IECtg*^ mice compared with the control WT mice (Fig. 1l).

Chronic inflammation plays a critical role in the occurrence and development of obesity [40]. Proinflammatory cells such as M1 macrophages and Th1 cells are often found in the adipose tissues; on the opposite, certain anti-inflammatory cell types, including M2 macrophages and Tregs, are more abundant in nonobese individuals [40, 41]. Indeed, M2 and Treg cells were markedly increased in the adipose tissues of *huREG4*^*IECtg*^ mice, which were resistant to HFD-mediated obesity, whereas their levels were significantly decreased in *Reg4* KO mice, which were more sensitive to HFD-mediated obesity (Fig. 1m and n). Thus, gut-derived Reg4 (REG4 in humans) is involved in sensitivity to HFD-mediated obesity.

### Reg 4 is related to IL-35^+^ cell accumulation

**Fig. 2:**
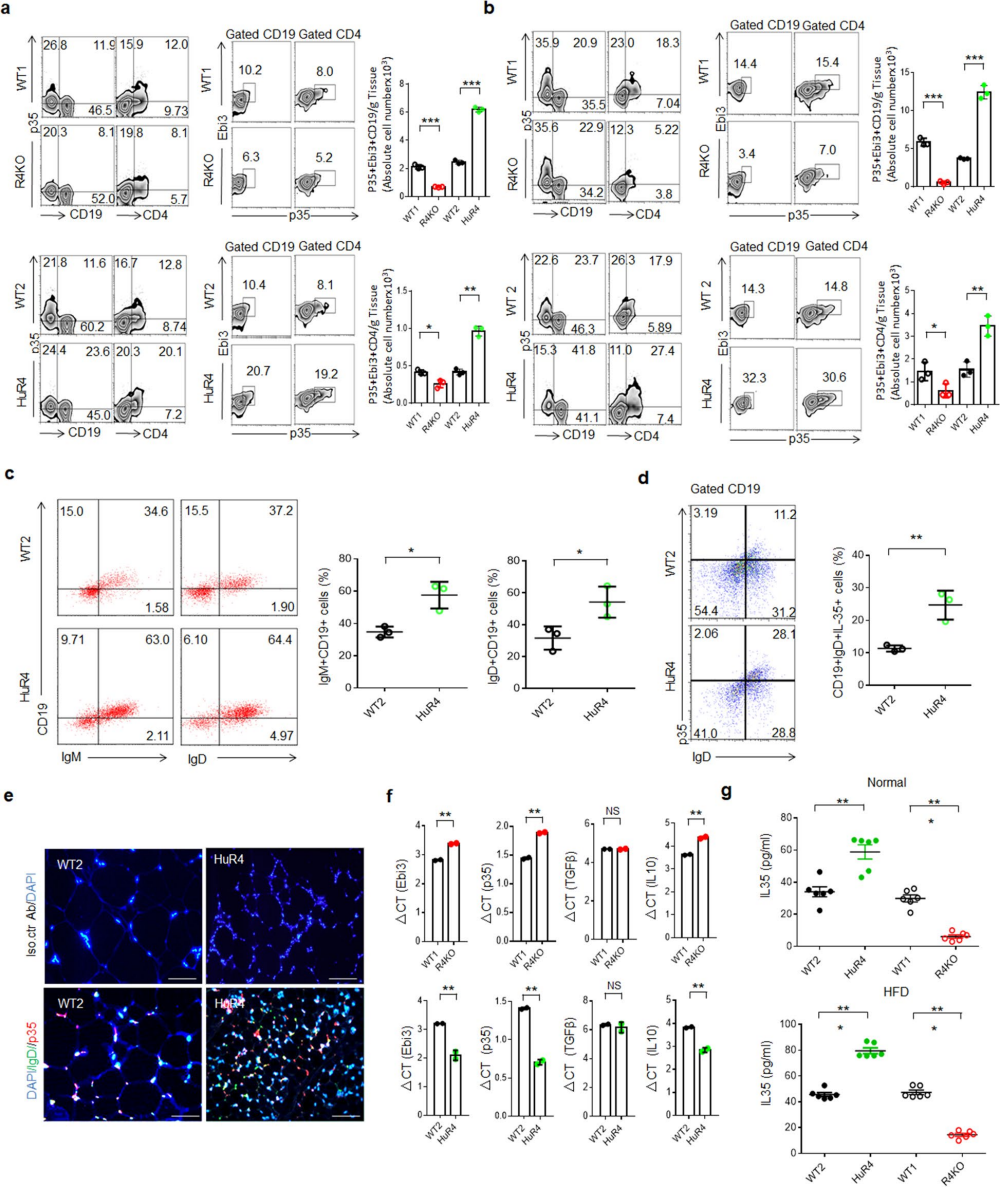
Reg 4 promotes the accumulation of IL-35^+^ cells in adipose tissues. **a** Flow cytometry of p35^+^CD19^+^, p35^+^CD4^+^, p35^+^Ebi3^+^CD19^+^, and p35^+^Ebi3^+^CD4^+^ cells in the fat pat adipose tissues of *Reg4* KO (R4KO) and control WT (WT1) mice and in *huREG4*^*IECtg*^ (HuR4) mice and their littermate controls (WT2) fed a HFD for 3 months. **b** Flow cytometry of p35^+^CD19^+^, p35^+^CD4^+^, p35^+^Ebi3^+^CD19^+^, and p35^+^Ebi3^+^CD4^+^ cells in the subcutaneous adipose tissues of *Reg4* KO (R4KO) and control WT (WT1) and in *huREG4*^*IECtg*^ (HuR4) mice and their littermate controls (WT2) fed a HFD for 3 months. **c** Flow cytometry of CD19^+^IgM^+^ and CD19^+^IgD^+^ cells in the subcutaneous adipose tissues of *huREG4*^*IECtg*^ (HuR4) mice and their littermate controls (WT2) fed a HFD for 3 months. **d** Flow cytometry of IgD^+^p35^+^ cells in the adipose tissues of *huREG4*^*IECtg*^ (HuR4) and control littermate WT mice (WT2) fed a HFD for 3 months. **e** Immunostaining of IgD^+^p35^+^ in the adipose tissues of *huREG4*^*IECtg*^ (HuR4) and control littermate WT mice (WT2) fed a HFD for 3 months. One representative result is shown from each group. *Iso. ctr Ab*, isotypic antibody. **f** qRT-PCR of IL-35 subunits (Ebi3 and p35), TGFβ and IL-10 in the adipose tissues of Reg4 KO (R4KO), WT (WT1) and *huREG4*^*IECtg*^ (HuR4) mice and their control WT littermates (WT2) fed a HFD for 3 months (mixed sample). **g** ELISA of IL-35 in *Reg4* KO (R4KO), WT (WT1) and *huREG4*^*IECtg*^ (HuR4), WT (WT2) mice with (HFD) or without (normal) HFD feeding for 3 months. The data in **a**, **b**, **c**, **d**, and **f** were from three independent experiments; the data in **g** were from one representative experiment. Scale bars = 40 μm; Student’s *t* test in all panels, mean ± SD; *p < 0.05, **p < 0.01, and ***p < 0.001; *NS*, no significance

We next attempted to identify factor(s) capable of altering the M2 and Treg cell proportions in adipose tissues. Anti-inflammatory cells such as Tregs and M2 macrophages can be induced by IL-10, TGFβ, and IL-35 [21, 42]. However, there are very few reports on IL-35 in adipose tissues. Here, we found that the numbers of both IL-35^+^ B and IL-35^+^ CD4 cells were markedly reduced in the adipose tissues (fat pad and subcutaneous adipose tissues) of *Reg4* KO mice, whereas these IL-35^+^ cells were significantly increased in *huREG4*^*IECtg*^ mice (Fig. 2a, b; Fig. S2). These increased IL-35^+^ B cells were identified to be IgM^+^ and IgD^+^ B cells (Fig. 2c, d). The accumulation of IL-35^+^IgD^+^ B cells in the adipose tissues of *huREG4*^*IECtg*^ mice was further confirmed using immunohistochemical staining (Fig. 2e). The transcript levels of the IL-35 subunits Ebi3 and p35 were also higher in the adipose tissues of *huREG4*^*IECtg*^ mice and lower in those of *Reg4* KO mice compared with their respective controls (Fig. 2f). Cytokine analyses also showed higher levels of IL-35 in the peripheral blood of *huREG4*^*IECtg*^ mice and lower levels in *Reg4* KO mice (Fig. 2g). Thus, these data showed marked IL-35^+^ Breg accumulation in the adipose tissues of *huREG4*^*IECtg*^ mice.

Breg cells have multiple subsets, including immature and mature B cells. We next analyzed markers expressed on different Breg cell types, including IgM, IgD, IL-10, CD1d, CD5, CD11, CD21/CD35, CD23, CD24, CD25, CD69, CD72, CD138, CD40, and CD86 [9, 43, 44]. The increased IL-35^+^ Breg cell proportions in the adipose tissue of *huREG4*^*IECtg*^ mice were identified as CD19^+^IgM^+^IgD^+^IL10^+^CD1d^high^CD5^low^CD11b^low^CD21/CD35^Low^CD23^Low^CD25^Low^

CD72^low^CD69^-^CD138^low/-^CD40^low/-^CD86^low/-^ cells (Fig. S3), which were different from IgG-producing Breg cells [45, 46] but similar to IgM^+^IgD^+^ Bregs [43] in adipose tissues; however, the expression levels of some markers were different.

Moreover, marked IL-35^+^ cell accumulation was observed in the colon lamina propria (LP), Peyer’s patch (PP), and splenic tissues of *huREG4*^*IECtg*^ mice, and the proportions of IL-35^+^ cells in these tissues were markedly decreased in *Reg4* KO mice (Fig. S4a-e). Immunostaining also revealed an increased number of IL-35^+^ cells in the colonic tissues of *huREG4*^*IECtg*^ mice but fewer IL-35^+^ cells in those of *Reg4* KO mice (Fig. S4c). Since the p35 and p40 subunits can form IL-12 and Ebi3 and p28 form IL-27 [21], we also detected the IL-27 and IL-12 cytokines. Higher levels of the IL-35 cytokine but not IL-27 and IL-12 were observed in the colon tissues of *huREG4*^*IECtg*^ mice (Fig. S4f), indicating that the IL-35 subunits p35 and Ebi3 did not affect the expression of IL-27 and IL-12. Taken together, these data show that the Reg4 expressed in gut epithelial cells can promote the accumulation of IL-35^+^ cells in adipose tissue as well as in gut tissues and peripheral organs.

### Reg4-associated gut microbiota/metabolites are related to IL-35^+^ cells

**Fig. 3:**
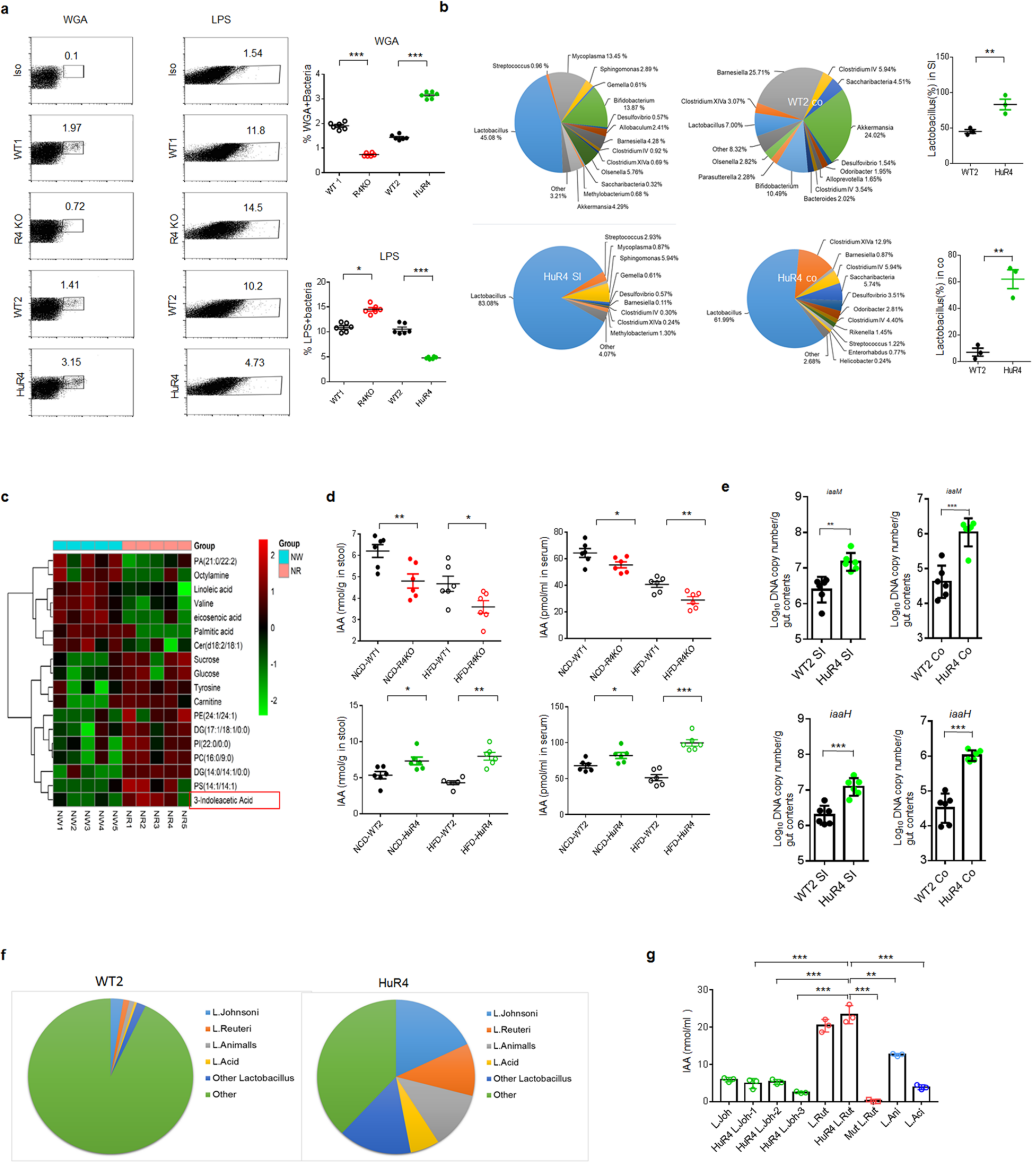
Reg4 affects the composition and metabolites of the gut microbiota. **a** Flow cytometry of WGA^+^ and LPS^+^ bacteria in fresh feces of *Reg4* KO (R4KO) and control WT (WT1) mice and in *huREG4*^*IECtg*^ (HuR4) mice and their littermate controls (WT2). *Iso*, isotypic control. **b** The proportions of gut bacteria after the 16S rRNA sequencing of the gut microbiota in pooled ileal (SI) and colonic (Co) samples from WT and *huREG4*^*IECtg*^ (HuR4) mice (three independent experiments, *n* = 5 mice, 7–8 weeks old, male). **c** LC-MS/MS analyses of peripheral blood of *huREG4*^*IECtg*^ (NR1-NR5) mice and their control littermates (NW1-NW5) fed normal chow (*n* = 5). **d** Analyses of IAA in the fresh stools and peripheral blood of WT (WT1), *Reg4* KO (R4KO), *huREG4*^*IECtg*^ (HuR4) mice, and their control littermates (WT2) fed (HFD-WT, HFD-R4KO, or HFD-HuR4) or not fed (NCD-WT, NCD-R4KO, or NCD-HuR4) a HFD for 3 months. **e** QPCR of the *iaaM* and *iaaH* genes in the ileal (SI) and colonic (Co) samples from *huREG4*^*IECtg*^ mice (HuR4) and their control littermates (WT2) (*n* = 6). Standard curves were prepared from serial dilutions of iaaM or iaaH, which were amplified in the guts of mice. **f** Proportion of different lactobacilli in the colons of WT (WT2) and *huREG4*^*IECtg*^ mice (HuR4). **g** IAA levels in the supernatants of different *Lactobacillus* species. *L. Joh*, *Lactobacillus johnsonii* (BNCC, China); *HuR4L.Joh-1*, 2, 3, three different strains isolated from *huREG4*^*IECtg*^ mice; *HuR4L. Rut*, an isolated strain of *Lactobacillus reuteri* from *huREG4*^*IECtg*^ mice; *L. Rut*, *Lactobacillus reuteri* (BioGaaia, Sweden); *L. Ani*, *Lactobacillus animalis* (BNCC, China); L.Aci, *Lactobacillus acidophilus* (BNCC, China); *Mut Lut*, *iaaM* deleted *Lactobacillus reuteri*. The data are from three independent experiments. Student’s *t* test in all panels, mean ±SD. *p < 0.05, **p < 0.01, and ***p < 0.001

Gut microbiota/metabolites play a critical role in the formation of the immune system [1–3]. Reg4 can not only kill *E. coli* through a complement-dependent pathway [36] but also damage the bacterial cell wall [34, 35], implying that it might alter the gut microbiota. Indeed, flow cytometry showed increased proportions of wheat germ agglutinin (WGA)^+^ bacteria but reduced proportions of LPS^+^ bacteria in the fresh stool of *huREG4*^*IECtg*^ mice, whereas the opposite trends were observed in *Reg4* KO mice compared with their controls (Fig. 3a), indicating alterations in the proportions of Gram^+^ and Gram^−^ bacteria. Moreover, 16S ribosomal RNA (V3–V4 variable region) sequencing analyses of the ileum and colonic contents showed that the proportion of *Lactobacillus* was markedly higher in *huREG4*^*IECtg*^ mice than in the control mice (Fig. 3b; Fig. S5).

Since gut microbiota/metabolites play a critical role in forming the immune system and maintaining gut immune homeostasis [1, 2], we hypothesized that the accumulation of IL-35^+^ cells in adipose and gut tissues was derived from changes in the gut microbiota. To establish the relationship between the gut microbiota and IL-35^+^ cells, we performed fecal exchange experiments. When *huREG4*^*IECtg*^ mouse feces were transferred into WT mice, the proportion of IL-35^+^ cells in the WT mice increased, whereas the transfer of *Reg4* KO mouse feces into WT mice reduced the number of IL-35^+^ cells in colonic LP tissues (Fig. S6a, b). Furthermore, the CD19^+^p35^+^ cell numbers were markedly increased in germ-free (GF) mice receiving *huREG4*^*IECtg*^ mouse feces but not in GF mice receiving *Reg4* KO mouse feces (Fig. S6c). The data also showed that equal amounts of bacteria were transferred to the different mice (Fig. S6e). All of these results suggest that Reg4-associated gut microbiota/metabolites play a role in the generation of IL-35^+^ cells.

### IAA can induce IL-35^+^ B cells in the presence of LPS

**Fig. 4:**
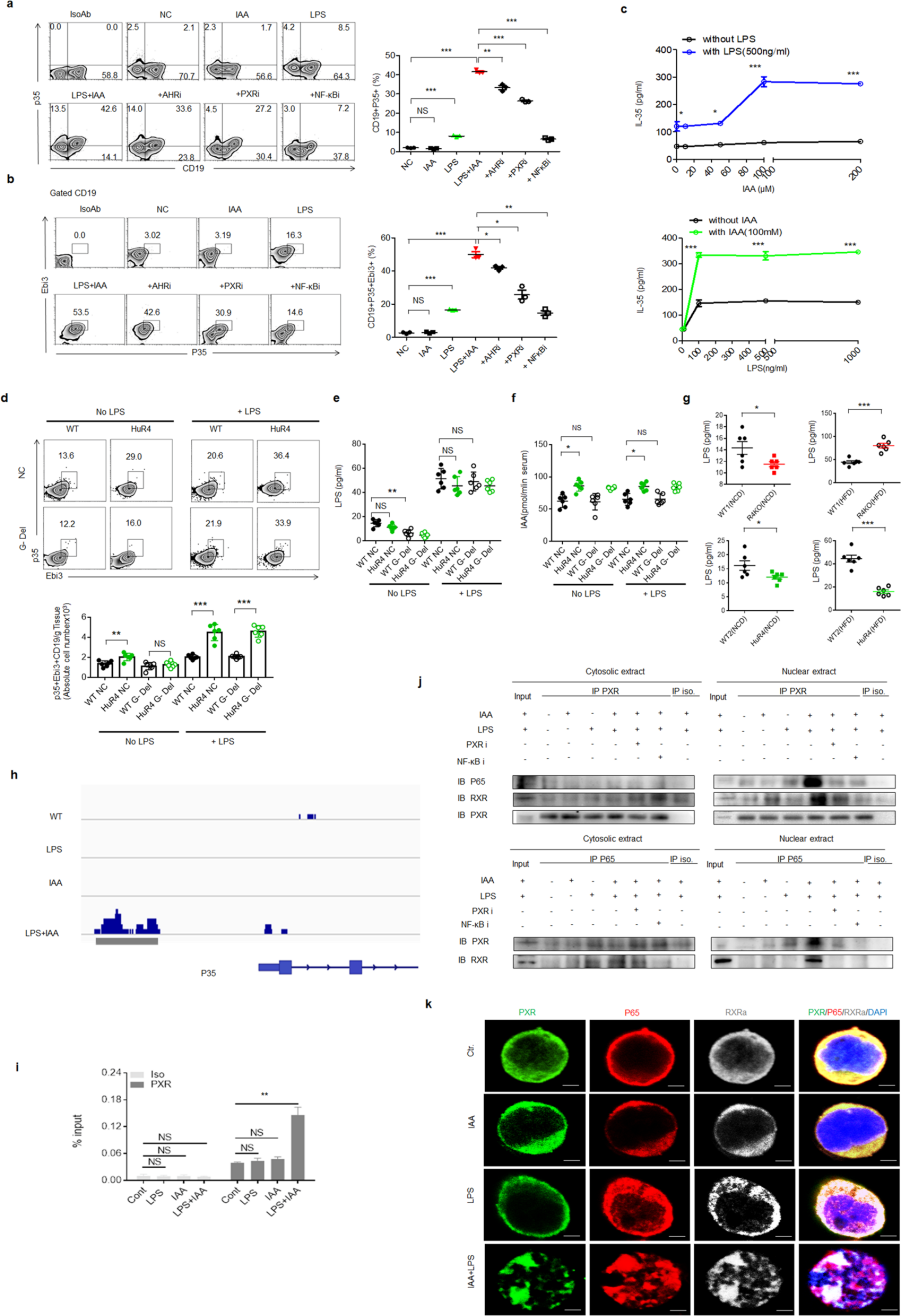
Together, IAA and LPS promote the binding of PXR to NFκB p65 and RXR. **a** Flow cytometry of p35^+^CD19^+^ cells in the spleen after exposure to IAA, LPS, LPS + IAA, LPS + IAA + AhR inhibitor (+AHRi), LPS + IAA + PXR inhibitor (+PXRi), or LPS + IAA + NFκB inhibitor (+NFκBi) for 24 h. *IsoAb*, isotype control; *NC*, unstimulated control. **b** Flow cytometry of p35^+^Ebi3^+^CD19^+^ cells in the spleen after exposure to IAA, LPS, LPS + IAA, LPS + IAA + AHR inhibitor (+AHRi), LPS + IAA + PXR inhibitor (+PXRi), or LPS + IAA + NFκB inhibitor (+NFκBi) for 24 h. *IsoAb*, isotype control; *NC*, unstimulated control. **c** ELISA of IL-35 in the supernatants of spleen cells after exposure to different concentrations of IAA in the presence of LPS or different concentrations of LPS in the presence of IAA. **d** Flow cytometry of CD19^+^p35^+^Ebi3^+^ cells in the adipose tissues of Gram^−^ bacteria–deleted WT and *huREG4*^*IECtg*^ mice (HuR4) treated with or without LPS. **e** LPS levels in the blood of Gram^−^ bacteria-deleted WT and *huREG4*^*IECtg*^ mice (HuR4) treated with or without LPS. **f** IAA levels in the blood of Gram^−^ bacteria-deleted WT and *huREG4*^*IECtg*^ mice (HuR4) treated with or without LPS. **g** LPS concentrations in the sera of WT (WT1), *Reg4* KO (R4KO), and *huREG4*^*IECtg*^ (HuR4) mice and their littermate controls (WT2) fed (HFD) or not fed (NCD) a HFD for 3 months. **h** ChIP sequencing analyses of the B lymphocyte WEHI 231 B cells after exposure to LPS, IAA or LPS + IAA for 6 h. **i** ChIP-PCR of the PXR binding site on the promoter region of p35 in WEHI 231 B cells after exposure to LPS, IAA or LPS + IAA for 6 h. **j** Immunoblotting of p65, RXRa, and PXR in the cytosolic and nuclear extracts of immunoprecipitants with anti-PXR or anti-p65. *Iso*, isotypic antibody. **k** Immunostaining of PXR, p65, and RXRa in WEHI231 B cells after exposure to IAA + LPS. Ctr., no stimulation. The data in **a**, **b,** and **c** were from three independent experiments; the data in **d**–**g** were from one representative experiment. Student’s *t* test in **a**, **b, d, e, f, g, and i;** mean ± SD; analysis of variance in **c**. *p < 0.05, **p < 0.01, and ***p < 0.001*; NS*, no significance

We next investigated the factor(s) in gut microbiota/metabolites capable of inducing IL-35^+^ cell generation. IL-35 is comprised of the Ebi3 and p35 heterodimer subunits [21], each of which is encoded by separate chromosomes and regulated independently [47]. These subunits are targets of microbial Toll-like receptor (TLR) agonists [47]. LPS, a TLR4 agonist, indeed induced the generation of IL-35^+^ B cells (Fig. 4a). However, the levels of LPS was not related to the proportion of IL-35^+^ cells in *huREG4*^*IECtg*^ and *Reg4 KO* mice (Fig. 4g), implying that increased IL-35^+^ cells in *huREG4*^*IECtg*^ are not induced by LPS alone. Other researchers also found that B cell differentiation into IL-35^+^ Bregs required costimulation with both TLR4 and CD40L [9]. To find a potential metabolite of gut microbiota, which is related to IL-35^+^ cells, we performed LC-MS/MS analyses. The levels of IAA were markedly increased in the peripheral blood and fresh stool of *huREG4*^*IECtg*^ mice; whereas lower levels of IAA were observed in *Reg4* KO mice as compared with their respective control (Fig. 3c, d). QPCR analyses also showed higher levels of *iaaM* and *iaaH* genes, which promote the generation of IAA in *huREG4*^*IECtg*^ mice [11] (Fig. 3e). Further studies showed a significantly higher proportion of *Lactobacillus reuteri* in the ilea and colons of *huREG4*^*IECtg*^ mice (Fig. 3f), which thereby increased production of IAA (Fig. 3g). Moreover, the blood levels of IAA were higher in WT or GF mice that received *huREG4*^*IECtg*^ mouse feces (Fig. S6d). All of these suggest that IAA might be a potential candidate to induce IL-35^+^ cells.

IAA is a potent bioactive metabolite that activates the pregnane X receptor (PXR) or AhR [11]. Since multiple potential PXR and AhR binding sites exist on the promoter regions of p35 and Ebi3, two subunits of IL-35 (https://biogrid-lasagna.engr.uconn.edu/lasagna_search/), we analyzed the ability of IAA to induce the generation of IL-35^+^ cells. However, IAA alone did not significantly increase the proportion of IL-35^+^ cells *in vitro* (Fig. 4a). We next evaluated the use of IAA together with LPS to induce the generation of IL-35^+^ cells, and IAA and LPS applied in combination induced markedly higher proportions of IL-35^+^ B cells than LPS or IAA alone *in vitro* (Fig. 4a, b). Furthermore, a marked dose response was observed after exposure to different concentrations of IAA in the presence of LPS (Fig. 4c). Since IAA can activate PXR or AhR [11], we observed the effects of PXR and AhR inhibitors on the generation of IL-35^+^ cells. Both the PXR inhibitor and the AhR inhibitor suppressed both the IAA- and LPS-mediated generation of IL-35^+^ B cells, but the PXR inhibitor was stronger (Fig. 4a, b), suggesting that PXR plays a main role in inducing the generation of IL-35^+^ cells. Moreover, the NFκB inhibitor markedly suppressed the generation of IL-35^+^ B cells (Fig. 4a, b). Notably, IAA together with LPS did not effectively induce the generation of IL-35^+^ T cells *in vitro* (Fig. S7), implying that IL-35^+^CD4^+^ T cells were derived from the IL-35 produced by IL-35^+^ B cells *in vivo* [21, 28]. Thus, IAA induced the generation of IL-35^+^ B cells in the presence of LPS*.*

We next investigated whether the endogenous LPS levels in the *huREG4*^*IECtg*^ mouse circulation and tissues were sufficient to prime B cells *in vivo*. To demonstrate this, gentamicin was used to kill the Gram-negative bacteria in *huREG4*^*IECtg*^ mice, thereby eliminating LPS-producing bacteria [48], and then examined the IL-35^+^ B cells in adipose tissues. No differences were observed between WT and *huREG4*^*IECtg*^ mice after the deletion of Gram-negative bacteria (Fig. 4d), indicating that the increased generation of IL-35^+^ B cells in *huREG4*^*IECtg*^ mice require LPS. Notably, more IL-35^+^ B cells were rescued by LPS in *huREG4*^*IECtg*^ mice (Fig. 4d). LPS was not detected in the plasma after the deletion of Gram-negative bacteria, but plasma IAA was detected (Fig. 4e, f). Certain levels of plasma LPS have also been observed in humans and animals with metabolic syndrome [37, 49, 50] (Fig. 4g). All of these results suggest that LPS is necessary for the production of IL-35^+^ cells. Thus, IAA can induce IL-35^+^ B cells in the presence of LPS *in vivo*.

### PXR and TLR4 are required for the mediation of IL-35^+^ B cells by IAA and LPS

p35, a subunit of IL-35, can increase the proportion of IL-35–expressing Breg cells [20]. ChIP sequencing and ChIP-PCR showed marked enrichment of PXR in the promoter region of p35 (a subunit of IL-35) in WEHI B cells treated with both IAA and LPS but not in those treated with IAA or LPS alone (Fig. 4h, i). After exposure to IAA together with LPS, PXR expression was markedly increased in both the cytoplasm and the nucleus (Fig. S8a), suggesting that IAA works together with LPS to promote the activity of PXR. In cells exposed to IAA and LPS, both NFκBp65 and retinoic X receptor (RXR) were observed in the nucleus (Fig. S8a). PXR exerts its transcriptional regulatory functions by dimerizing with RXR [51, 52]. We indeed observed increased binding between PXR and RXR in the nucleus after exposure to IAA together with LPS but not IAA or LPS alone (Fig. 4j; Fig. S8b). This binding was impeded by PXR and NFκB inhibitors (Fig. 4j), suggesting that both PXR and NFκB are necessary for the complex. We also performed immunoprecipitation (IP) assays with an anti-NFκB p65 antibody, and the results showed a marked increase in the expression of PXR in the nucleus after exposure to IAA together with LPS (Fig. 4j). However, IAA and LPS in combination also promoted the binding of NFκBp65 to RXR (Fig. 4j), suggesting that NFκBp65 binds not only to PXR but also to RXR. Immunostaining assays also showed increased binding among PXR, NFκB, and RXR after exposure to LPS and IAA together (Fig. 4k). Thus, complexes composed of PXR, NFκB, and RXR are necessary for the expression of IL-35. In addition, inactive PXR is predominantly sequestered in the cytoplasm [53, 54] by cytoplasmic androstane receptor (CAR) retention protein and heat shock protein 90 complexes [55, 56]. IP performed using anti-PXR also revealed the binding of PXR with CAR in the cytosolic extract (Fig. S8c). Furthermore, this binding was decreased in the cytoplasm after exposure to IAA or IAA + LPS (Fig. S8c), indicating an increase in the level of active PXR. Immunostaining also showed increased PXR levels in the cytoplasm and nucleus after treatment with both IAA and LPS (Fig. S8d). All of these results suggest that IAA and LPS in combination can promote the entry of PXR into the nucleus to promote IL-35 expression by binding with NFκBp65 and RXR.

**Fig. 5:**
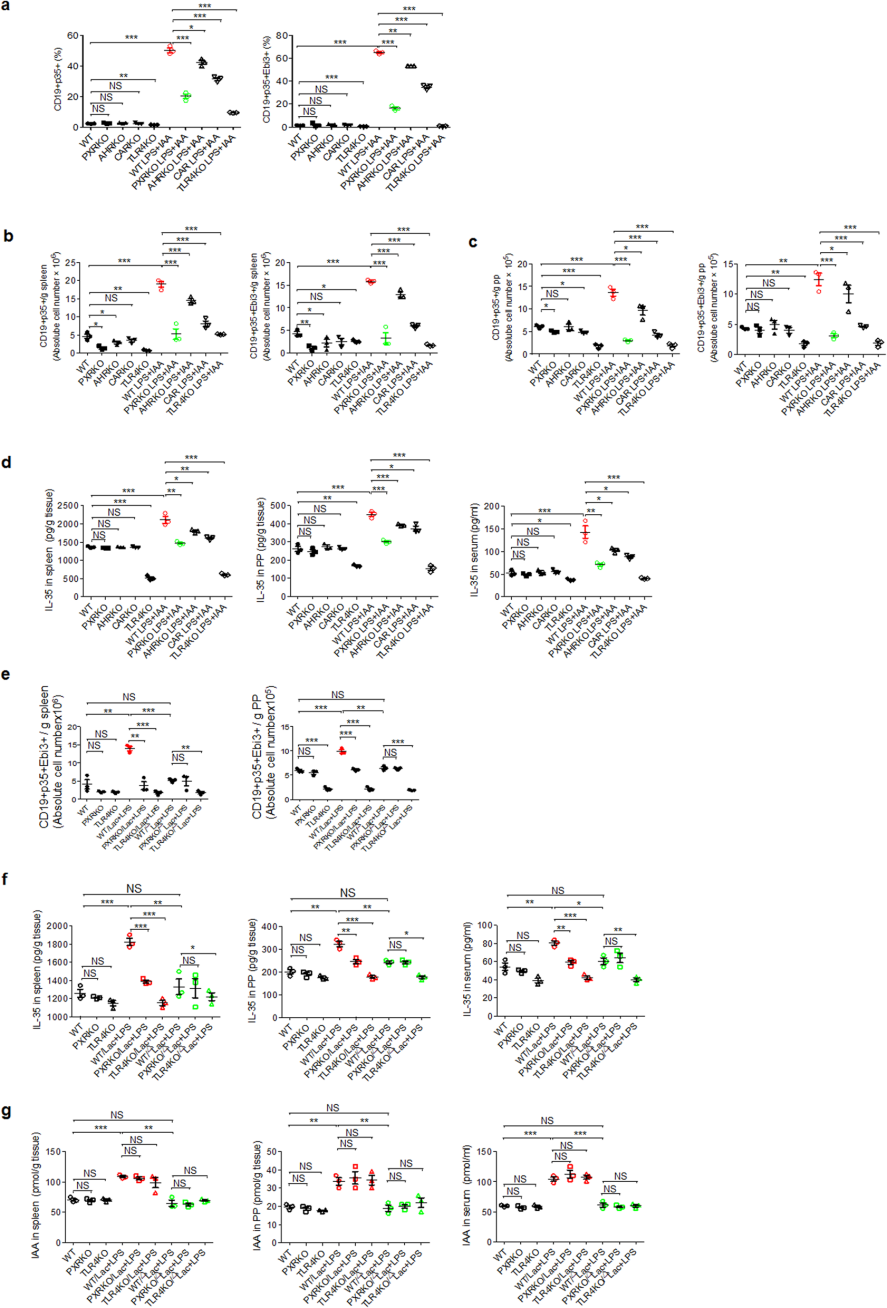
The mediation of IL-35^+^ B cells by IAA and LPS requires PXR and TLR4. **a** Percentages of CD19^+^p35^+^ and CD19^+^p35^+^Ebi3^+^ cells in the spleens of WT, *PXR* KO, *TLR4* KO, *CAR* KO, or *AHR* KO mice with or without exposure to LPS + IAA. **b** Absolute cell numbers of CD19^+^p35^+^ and CD19^+^p35^+^Ebi3^+^ cells in the spleens of WT, *PXR* KO, *TLR4* KO, *CAR* KO, and *AHR* KO mice treated with or without LPS + IAA. **c** Absolute numbers of CD19^+^p35^+^ and CD19^+^p35^+^Ebi3^+^ cells in the PPs of WT, *PXR* KO, *TLR4* KO, *CAR* KO, and *AHR* KO mice treated with or without LPS + IAA. **d** ELISA of IL-35 in the spleens, PPs, and sera of WT, *PXR* KO, *AHR* KO, *CAR* KO, and *TLR4* KO treated mice with or without LPS + IAA. **e** Absolute numbers of CD19^+^p35^+^Ebi3^+^ cells in the spleens and PPs of WT, *PXR* KO, and *TLR4* KO mice infused with or without *Lactobacillus* or *Lactobacillus*^*ΔiaaM*^ (^Δ^Lac). **f** ELISA of IL-35 in the spleens, PPs, and sera of WT, *PXR* KO, and *TLR4* KO mice infused with or without *Lactobacillus* or *Lactobacillus*^*ΔiaaM*^ (^Δ^Lac). **g** IAA levels in the spleens, PPs, and sera of WT, *PXR* KO, and *TLR4* KO mice injected with or without *Lactobacillus* or *Lactobacillus*^*ΔiaaM*^ (^Δ^Lac). The data in each panel were from three independent experiments. Student’s *t* test in all panels, mean ± SD. *p < 0.05, **p < 0.01, and ***p < 0.001*, NS*, no significance

To further demonstrate that IAA- and LPS-mediated IL-35 cells are dependent on PXR and NFκBp65, we employed *PXR* KO and *TLR4* KO mice (TLR4 can activate NFκBp65). Since active PXR is regulated by the CAR retention protein [55, 56], the effects of *CAR* KO on IL-35^+^ cells were also observed. We also observed *AhR* KO mice, in which AhR was potentially recognized by IAA [11]. Splenic cells from WT, *PXR* KO, *TLR4* KO, *CAR* KO, and *AhR* KO mice were cultured in medium supplemented with both IAA and LPS. IAA and LPS in combination did not effectively induce the generation of IL-35^+^ B cells from the splenic cells of *PXR* KO or *TLR4* KO mice, whereas the splenic cells from WT mice produced more IL-35^+^ B cells in the presence of both IAA and LPS than the cells treated with IAA or LPS alone (Fig. 5a; Fig. S9a). Notably, *CAR* KO also had significant effects on the generation of IL-35^+^ B cells compared with that in WT mice (Fig. 5a; Fig. S9a), consistent with the above findings (Fig. 4). After the *in vivo* injection of IAA plus LPS into *PXR* KO, *CAR* KO, *TLR4* KO, and *AhR* KO mice, the generation and accumulation of IL-35^+^ cells was promoted in WT mice but not in *PXR* KO or *TLR4* KO mice (Fig. 5b, c; Fig. S9b, d). Higher levels of IL-35 were detected in the spleens, Peyer’s patches (PPs), and peripheral blood of WT mice than in those of *PXR* KO and *TLR4* KO mice (Fig. 5d), and less IL-35 accumulation was observed in the spleens, PPs, and peripheral blood of *CAR* KO mice (Fig. 5d). Immunostaining also further confirmed the increased IL-35^+^CD19 cell proportions in the spleens of WT mice injected with IAA and LPS (Fig. S9c). In addition, *AhR* KO also affected the generation of IL-35^+^ cells *in vitro* and *in vivo* to some degree (Fig. 5a–d), suggesting that AhR is partially involved in the IAA + LPS-mediated generation of IL-35^+^ cells.

Since the *Lactobacillus* proportions were significantly higher in the ilea and colons of *huREG4*^*IECtg*^ mice, we isolated one dominant *Lactobacillus* strain, *Lactobacillus reuteri,* which can produce IAA, from the fresh stools *of huREG4*^*IECtg*^ mice (Fig. 3g). We also generated a mutant *Lactobacillus* species (Lactobacillus^*ΔiaaM*^*)* that could not produce IAA (Fig. 3g). When these Lactobacilli or Lactobacilli^*ΔiaaM*^ were infused into mice, the Lactobacilli induced the production of IL-35 in the presence of LPS, whereas the lactobacilli^*ΔiaaM.*^ did not (Fig. 5e; Fig. S10a, b)*.* Notably, lactobacilli did not induce the generation of IL-35^+^ B cells in *PXR* KO or *TLR4* KO mice (Fig. 5e; Fig. S10a, b), and higher levels of the IL-35 cytokine were observed in the spleens, PPs, and peripheral blood of WT mice compared with the *PXR* KO and *TLR4* KO mice infused with Lactobacillus (Fig. 5f). Unlike Lactobacillus, the infusion of Lactobacillus^*ΔiaaM*^ did not affect the levels of IAA in the spleens, PPs, or peripheral blood of the mice (Fig. 5g). Thus, IAA generated by Reg4-associated Lactobacillus promotes the generation of IL-35^+^ cells in WT mice but not in *PXR* KO or *TLR4* KO mice.

### Reg4 promotes resistance to HFD-induced obesity via IL-35

**Fig. 6:**
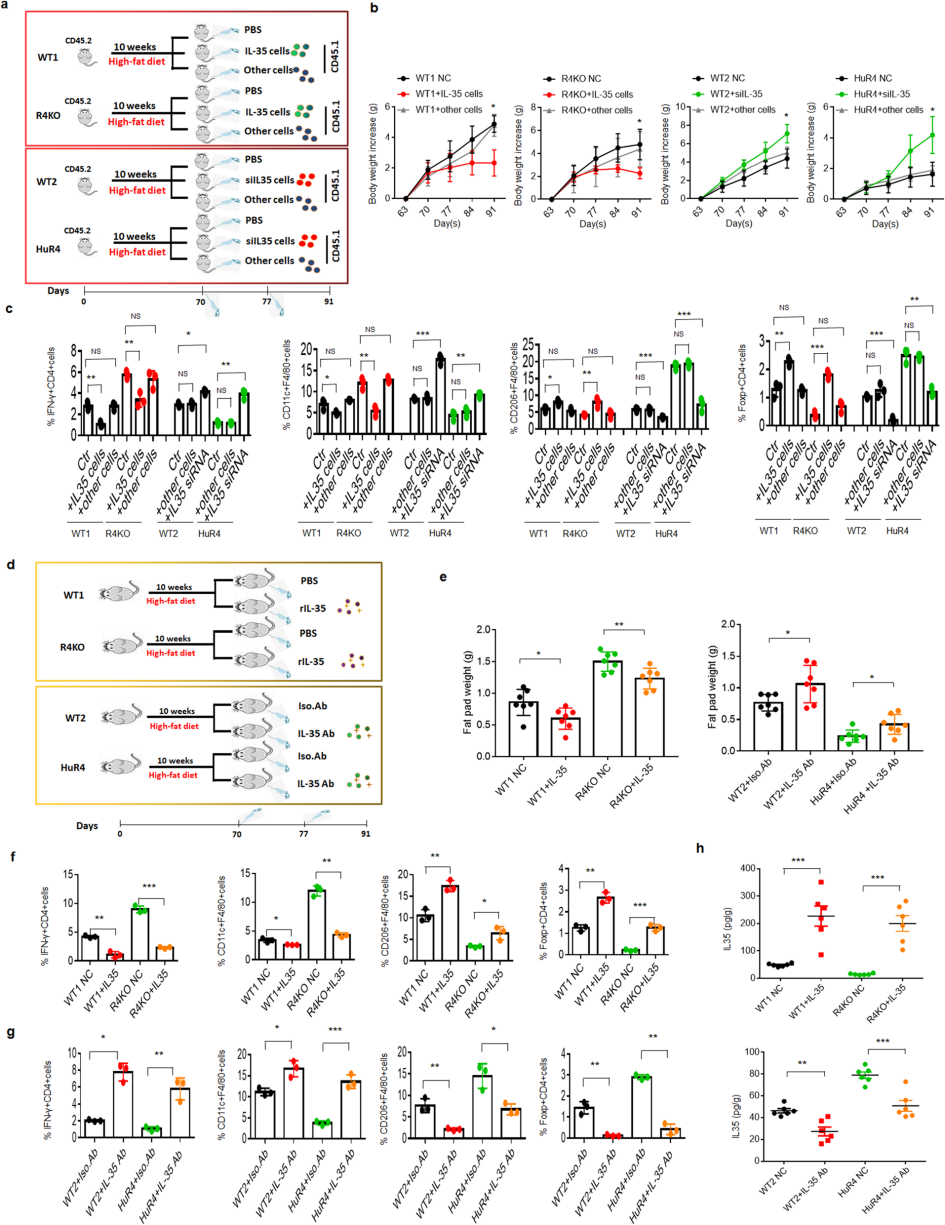
IL-35 promotes resistance to HFD-induced obesity. **a** Study design of the *in vivo* mouse experiment. **b** Changes in the body weights of mice fed a HFD. Mice were fed a HFD for 10 weeks and then transplanted with IL-35^+^ B cells (+IL-35 cells) treated with (+IL-35 siRNA) or without siRNA. Other cells, isolated B cells from spleen. **c** Flow cytometry of F4/80^+^CD11C^+^, F4/80^+^CD206^+^ cells, IFNγ^+^CD4^+^, Foxp3^+^CD4^+^ in the fat pads of WT1 and *Reg4* KO (R4KO) mice and WT2 and *huREG4*^*IECtg*^ (HuR4) mice fed a HFD and then transplanted with IL-35^+^ B cells (+IL-35 cells) treated with (+IL-35 siRNA) or without siRNA (*n* = 3). Other cells, isolated B cells from spleen. **d** Study design of the *in vivo* mouse experiment. **e** Changes in the fat pad weights of mice fed a HFD for 10 weeks and treated with or without rIL-35 and IL-35 neutralizing antibodies. The mice were fed a HFD for 10 weeks and then injected with rIL-35 or IL-35 neutralizing antibodies via their inguinal fat pad tissues. WT (WT1) and Reg4KO (R4KO) mice treated with PBS (NC) or rIL-35 (+IL-35); WT (WT2) and *huREG4*^*IECtg*^ (HuR4) mice treated with an isotype antibody (+Iso. Ab) or a IL-35 neutralizing antibody (+IL-35 Ab) (*n* = 7, per group). **f, g** Flow cytometry of F4/80^+^CD11C^+^, F4/80^+^CD206^+^, IFNγ^+^CD4^+^, and Foxp3^+^CD4^+^ cells in the fat pad tissues of WT (WT1) and *Reg4* KO mice (R4KO) treated with or without rIL-35 in (**f**) and in WT (WT2) and *huREG4*^*IECtg*^ mice (HuR4) treated with or without an anti-IL-35 neutralizing antibody in (**g**) (*n* = 3). **h** ELISA of IL-35 in the fat pad tissues of mice injected with rIL-35- or IL-35-blocking antibodies. The data in **b**, **c**, **f**, and **g** were from three independent experiments; the data in **e** and **h** were from one representative experiment. Analysis of variance in **b**; Student’s *t* test in the other panels, mean ± SD. *p < 0.05, **p < 0.01, and ***p < 0.001

We next assessed whether the Reg4-mediated resistance to HFD-induced obesity was dependent on IL-35. Since adoptive transfer studies using CD45.1^+^ and CD45.2^+^ congenic mice have been used to trace IL-35^+^ Bregs during inflammation [19] (Fig. S11), we generated CD45.1 IL-35^+^ B cells to determine the role of IL-35^+^ B cells in resistance to HFD-mediated obesity. Indeed, these IL-35^+^ B cells impeded the growth of adipose tissues, promoted insulin sensitivity and glucose tolerance, and reduced inflammation in the mouse adipose tissues (Fig. 6a–c; Fig. S12; Fig. S13a). However, the injection of IL-35–silenced B cells did not exert similar effects (Fig. 6a–c; Fig. S13a). We also assessed the fat pad weights and inflammation after the injection of rIL-35 or IL-35 neutralizing antibodies into the inguinal fat pad adipose tissues of the mice. The fat pad weights were markedly low in the mice injected with the rIL-35 but not in those injected with the IL-35 neutralizing antibody (Fig. 6d, e). Markedly decreased inflammation was also observed in adipose tissues, manifested as decreased proportions of IFNγ^+^ Th1 cells and increased proportions of Tregs and M2 macrophages in inguinal fat pads injected with rIL-35. On the other hand, increased adipose tissue inflammation was observed in the inguinal fat pads injected with IL-35 neutralizing antibodies, manifested as increased proportions of IFNγ^+^ Th1 cells and decreased proportions of Tregs and M2 macrophages (Fig. 6f, g; Fig. S13b, c). The IL-35 concentrations were also lower in the fat pad tissues of mice injected with the IL-35 antibody than in those injected with the control antibody (Fig. 6h). Taken together, these results show that Reg4 promotes resistance to HFD-induced obesity by increasing the level of IL-35.

### IAA levels are low in the peripheral blood of individuals with obesity

**Fig. 7:**
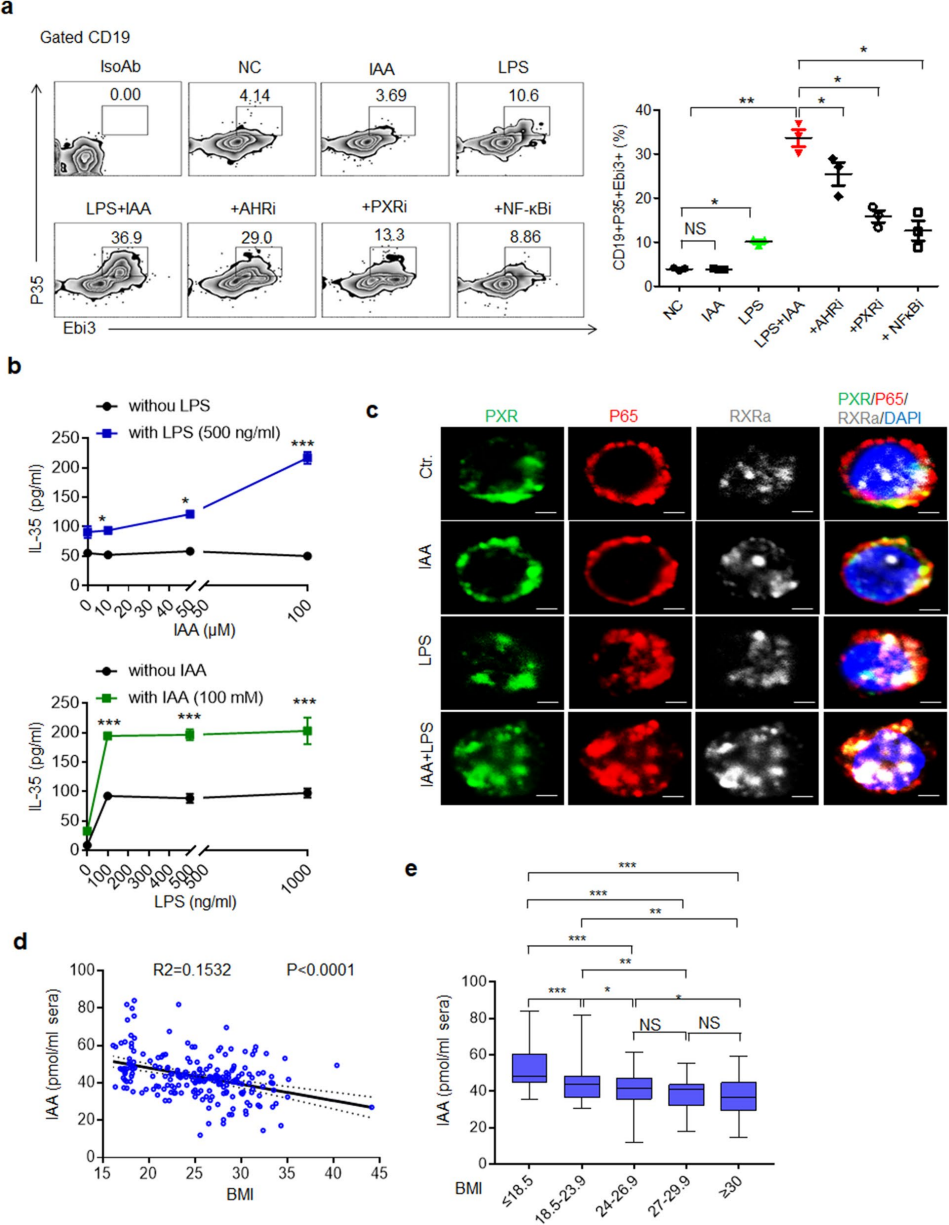
Levels of IAA in the peripheral blood of individuals with obesity. **a** Flow cytometry of p35^+^Ebi3^+^CD19^+^ cells in human peripheral blood cells after exposure to IAA, LPS, LPS+IAA, LPS + IAA + AhR inhibitor (+AHRi), LPS + IAA + PXR inhibitor+(PXRi), or LPS + IAA + NFκB inhibitor (+NFκBi) for 48 h. *IsoAb*, isotype control; *NC*, negative control. **b** ELISA of IL-35 in the supernatants of human peripheral blood cells after exposure to different concentrations of IAA with or without 500 ng/ml LPS or exposure to different concentrations of LPS with or without 100 mM IAA. **c** Immunostaining of PXR, p65, and RXRa in human peripheral blood cells after exposure to IAA + LPS for 6 h. *Ctr.*, no stimulation. **d** Negative correlation between the BMI and IAA levels in sera according as determined by linear regression analysis. *R*2 = 0.1532. **e** IAA levels in the peripheral blood of subjects from different groups, *BMI* ≤ 18.5 (*n* = 42), 18.5–23.9 (*n* = 41), 24–26.9 (*n* = 43), 27–29.9 (*n* = 42), and ≥ 30 (*n* = 40). The data in **a** and **b** were from three independent experiments. Student’s *t* test in **a**, mean ± SD; analysis of variance in **b**; Spearman nonparametric rank test for the correlation between the IAA level and BMI in **d**; and the Mann–Whitney U test in **e**, mean ±SEM. *p < 0.05, **p < 0.01, and ***p < 0.001; *NS*, no significance

Similar to those in mice, both IAA and LPS enhanced the generation of IL-35^+^ B cells in human peripheral blood cells *in vitro* (Fig. 7a). PXR and NFκB inhibitors had stronger suppressive effects on IL-35^+^ B cells mediated by both IAA and LPS than on those mediated by IAA or LPS alone (Fig. 7a). Furthermore, a marked dose response was observed after exposure to different concentrations of IAA in the presence of LPS (Fig. 7b). IAA together with LPS also promoted the entry of both NFκBp65 and RXR into the nucleus (Fig. 7c). Immunostaining showed increased binding among PXR, NFκBp65, and RXR after exposure to both LPS and IAA together (Fig. 7c), indicating that IAA and LPS in combination promote IL-35 expression by enhancing the binding of PXR to NFκB p65 and RXR in human B cells.

Obesity is also associated with the gut microbiota and metabolites [57, 58]. A systematic review indicated that probiotics capable of producing IAA may have beneficial effects on weight loss in overweight adults [59]. Thus, we investigated the relevance of gut microbiota–derived IAA to the body weights of 208 individuals with different body mass indices (BMIs). The concentrations of the microbiota metabolite IAA were lower in individuals with increased BMIs than in those with normal BMIs (Fig 7d). Indeed, individuals with obesity had lower IAA levels than the nonobese subjects (Fig 7e). Since the increased levels of IAA may induce the generation of IL-35^+^ cells in humans, we also detected the levels of IL-35 in the peripheral blood of overweight and obese individuals. Unexpectedly, the IL-35 levels were very low in all individuals with various BMIs. However, lower levels of IAA were indeed observed in overweight and obese individuals compared with the subjects with normal BMIs.

## Discussion

Here, we found Reg4 derived from gut epithelial cells to be related to resistance to HFD-mediated obesity. Marked IL-35^+^ cell accumulation was observed in the adipose tissues, gut tissues and other organs (e.g., spleen) of *huREG4*^*IECtg*^ mice, which were resistant to HFD-mediated obesity, whereas fewer IL-35^+^ cells were observed in *Reg4* KO mice than in their control WT littermates. The alteration of gut microbiota in *huREG4*^*IECtg*^ or *Reg4* KO mice was directly associated with IL-35^+^ cells. We also found the accumulation of IL-35^+^ cells to be related to the *Lactobacillus*-derived metabolite IAA. IAA induced the generation and accumulation of IL-35^+^ B cells in the presence of LPS by activating PXR. In addition, lower levels of IAA were observed in individuals with obesity than in nonobese subjects. These data suggest the existence of an axis among Reg4, gut microbiota, IAA/IL-35^+^ B cells, and resistance to HFD-mediated obesity.

We demonstrated that IAA together with LPS induced the generation of IL-35^+^ cells by activating PXR, NFκB, RXR, and CAR. The tryptophan metabolite IAA has been reported to act as an agonist of PXR [60]. Others also found that indole and indolic acid derivatives are potent bioactive metabolites that affect the intestinal barrier integrity and immune cells in mice by activating PXR [11, 61, 62]. Our results showed that IAA and LPS in combination promoted the activity of not only the transcription factor PXR but also RXR and CAR, which are necessary for inducing the expression of p35, a subunit of IL-35. Relationships and interactions among PXR, CAR, and RXR have been observed in multiple organs and tissues [63–65]. Several studies have also reported a relationship between IL-35 and NFκB; for example, B cells isolated from WT mice were shown to increase the expression of p35 and Ebi3 upon activation via TLR4 [9]. Another study found that influenza A virus (IAV)–induced IL-35 transcription was regulated by NFκB [66]. Additionally, direct chemical communication between intestinal symbionts and PXR can regulate mucosal integrity through a pathway that involves luminal sensing and signaling by TLR4 [61]. It also is completely possible for other same settings/milieu of external stimuli for the induction of IL-35^+^ B cells.

To date, a plethora of microbial intestinal catabolites of tryptophan (MICT), including indole (IND), IAA, tryptamine (TA), indole-3-pyruvate (IPY), indole-3-lactate (ILA), indole-3-acrylate (IAC), indole-3-propionate (IPA), skatole (3MI), indole-3-acetamide (IAD), indole-3-ethanol (IET), indole-3-aldehyde (IAID), and indole-3-acetaldehyde, have been identified [60]. These metabolites play an important role in inducing the differentiation of immune cells. For example, IAlD from Lactobacillus promotes AhR-dependent IL-22 transcription [11, 62, 67] and activates innate lymphoid cells (ILCs) [11], and AhR contributes to the transcriptional programming of IL-10-producing regulatory B cells [17]. We found that IAA could induce the generation of IL-35^+^ B cells by activating PXR receptors.

Lactobacilli are enriched in gut with Reg4. Reg4 can potentially induce damage to the bacterial cell wall to kill bacteria [34, 35]. Our previous studies also found that Reg4 could kill *E. coli* through a complement-dependent pathway [36]. Thus, it is possible that the killing of Reg4 on the other bacteria causes the increased Lactobacilli.

Our results suggest that lactobacilli producing IAA is negatively associated with the development of obesity. The levels of IAA are low in the peripheral blood of mice and humans with metabolic syndrome. Other researchers have also reported a strong negative correlation between the abundance of IAA and the body mass index (BMI) [7].

## Conclusion

Here, we found that the gut expression of Reg4 promotes resistance to HFD-induced obesity and the accumulation of IL-35^+^ cells in adipose tissues. We demonstrate that gut Reg4-associated microbiota such as *Lactobacillus* can promote the generation of IL-35^+^ B cells by producing IAA in the presence of LPS. Reg4 mediates resistance to HFD-induced obesity via IL-35, and the abundance of IAA is low in the peripheral blood of individuals with obesity. Finally, we demonstrated that IAA and LPS in combination mediate the production of IL-35^+^ B cells through PXR and TLR4. *PXR* KO or *TLR4* KO impairs the generation of IL-35^+^ B cells. Thus, together, IAA and LPS induce the generation of IL-35^+^ B cells through PXR and TLR4.

## Materials and methods

All reagents and oligos used in this study are listed in Supplementary Table S1.

### Mice

Four- to six-week-old male or female C57BL/6 mice were obtained from Nanjing Animal Center, Nanjing, China; *AhR* KO mice were obtained from the Third Military Medical University, Chongqing, China; *PXR* KO and *CAR* KO mice were obtained from the Chinese Academy of Inspection and Quarantine, Tianjin, China; *TLR4* KO mice were obtained from Shanghai Model Organisms Center, Shanghai, China. All experimental litters were bred and maintained under specific pathogen-free conditions at Nankai University. Experiments were carried out using age- and sex-matched mice. All procedures were conducted according to the Institutional Animal Care and Use Committee of the Model Animal Research Center. Animal experiments were approved by the Animal Ethics Committee of Nankai University.

C57BL/6 GF mice were generated by Beijing Animal Center. *Reg4*-deficient mice were generated by CRISPR/Cas-mediated genome engineering as previously described [36].

To generate transgenic (Tg) mice expressing human REG4 under the control of a 1.4-kb HD5 promoter, human REG4 cDNA was subcloned into the vector that contains two insulators for blocking other regulation function, the Tg Plasmid was digested with I-Ceu I, and the resultant 4.9-kb fragment was injected into fertilized oocytes of C57BL/6J mice in the Nanjing Biomedical Research Institute of Nanjing University. Then, the zygotes were transferred into the oviduct of pseudopregnant ICR females at 0.5 dpc. F0 mice was birthed after 19~21 days of transplantation.

The Funder Tg mice were identified using a standard PCR-based genotyping procedure with the following primers: HD5-REG4-tF1, 5’-gggatcttgagaacaaaggcagtc-3’ and HD5-REG4-tR1, 5’-TCAGACCAGTTCCTCAGCTTCCT-3’, yield a 338-bp product; HD5-REG4-tF2, 5’-ggttggctataaagaggtcatcag-3’and HD5-REG4-tR2, 5’-GCTGTCCCTCTAGCGAGATC-3’, yield a 250-bp product; and 42, 5’-CTAGGCCACAGAATTGAAAGATCT-3’ and 43, 5’-GTAGGTGGAAATTCTAGCATCATCC-3’, yield a 342-bp product from wild-type, which amplify the sequence at the junction of the HD5 promoter and REG4 gene.

### Human samples

For the collection of human serum, 208 adult participants, among which 42 with a BMI < 18.5 kg/cm^2^, 41 with a BMI of 18.5–24 kg/cm^2^, 43 with a BMI of 24–27 kg/cm^2^, 42 with a BMI of 27–30 kg/cm^2^ and 40 with a BMI ≥ 30 kg/cm^2^, were selectively recruited. More than half (54.3%) of the included patients were males, and the mean age was 42 years (*SD* = 13 years). The mean BMI was 25.05 ± 5.07 kg/cm^2^. All participants were free of acute stress conditions such as fever and diarrhea. Height and weight were measured to the nearest 0.1 cm and 0.1 kg without shoes or heavy clothing using a calibrated stadiometer (GL-310, Seoul, Korea). Participants were instructed to fast for ≥ 12 h before blood sampling the next morning. This study was conducted with approval from the Institutional Review Boards of Nankai University, Tianjin Union Medical Center, and Tianjin First Central Hospital. Participants were recruited from the health screening centers of Tianjin Union Medical Center and Tianjin First Central Hospital. All participants provided written informed consent.

### Mouse models

For the HFD model, 6- to 8-week-old male and female mice and their control littermates were fed a HFD (D12492, 26.2% protein, 26.3% carbohydrate, and 34.9% fat) or a control diet (D12450B), which was purchased from Research Diets, Inc. (New Jersey, USA).

For microbiota transplantation, 6- to 8-week-old mice were treated with pan-antibiotics (ampicillin (A, 1 g/l, Sigma), vancomycin (V, 0.5 g/l), neomycin sulfate (N, 1 g/l), and metronidazole (M, 1 g/l)) via their drinking water. Water containing the antibiotics was exchanged every 3 days. To confirm the elimination of bacteria, stool was collected from antibiotic-treated and untreated mice and cultured under anaerobic and aerobic conditions. The bacteria were counted under a microscope. Then, the cecal contents of detergent-treated mice or 1 × 10^9^ bacteria were suspended in 1 ml of PBS with 30% glycerol. The mice were removed from the isolator and orally administered 200 ml of the fecal suspension or bacteria made using glycerol stocks. For the *in vivo* administration of IAA together with LPS, the mice were randomly divided into 4 groups: normal group (intraperitoneally (i.p) with 0.2 ml of PBS only), IAA group (500 mg/kg IAA diluted in DMSO), LPS group (2 mg/kg O111:B4 in 0.2 ml of PBS), and IAA plus LPS group (500 mg/kg IAA and 2 mg/kg O111:B4). After administration for 24 h, tissues were isolated for further analyses. For Gram-negative bacterial deletion, mice were fed gentamicin (1 g/l, Sigma, for Gram-negative bacteria) for 1 week, and the deletion of Gram-negative bacteria was then confirmed. For the systemic adoptive transfer of B cells, mice were fed a HFD for 10 weeks and then intravenously injected with IL-35^+^ B cells treated with or without IL-35 siRNA (2 × 10^6^ cells/mouse/week, twice). IL-35^+^ B cells from CD45.1 mice were generated *in vitro* and isolated using flow cytometry. For the assessment of inguinal fat pad tissues, mice were fed a HFD for 10 weeks and then subcutaneously (s.c.) administered rIL-35 (1117574, Peprotech, 10 ng/mouse/week, twice) or an anti-IL-35 neutralizing antibody (C18.2, eBioscience™, 10 μg/mouse/week, twice) via their inguinal fat pad tissues. After 3 weeks, the tissues were evaluated.

### Ex vivo stimulation

For *ex vivo* stimulation, mouse splenic cells or human peripheral blood cells (approved by the Institute Research Ethics Committee of Nankai University, permit no: 200828), were collected, after which 5 × 10^6^ cells per well were seeded into a 24-well plate and then stimulated with IAA (100 μM), LPS (100 ng/ml), or IAA (100 μM) plus LPS (100 ng/ml) with or without a PXR inhibitor (10 μM), AhR inhibitor (10 μM), or NFκB inhibitor (10 μM) for 48 h.

For WEHI231 B cell stimulation, WEHI231 B cells were seeded in 24-well plates, stimulated with IAA and LPS with or without a PXR inhibitor or NFκB inhibitor for 3 or 6 h, and then harvested for WB, ChIP-SEQ, and ChIP-PCR analyses.

### Metabolism experiments

For the assessment of glucose tolerance and insulin sensitivity, baseline blood glucose levels were measured after 5 h of fasting using a Nova Max Plus GlucoseMeter. Mice were then i.p. injected with glucose (2 g/kg) in sterile PBS or with insulin (0.5 U/kg) (Sigma, St. Louis, Missouri), and their blood glucose levels were measured at different times after injection.

### Gut microbiome analyses

Gut microbiota were analyzed by Majorbio Biotechnology Company (Shanghai, China) using primers targeting the V3–V4 regions of 16S rRNA. After the PCR amplification of each sample, the amplicons were purified using the QIAquick PCR purification kit (Qiagen, Valencia, CA, USA), quantified, normalized, and then pooled in preparation for emulsion PCR followed by sequencing using titanium chemistry (Roche, Basel, Switzerland) according to the manufacturer’s protocol. Operational taxonomic unit (OTU) analysis was performed as follows: sequences were processed (trimmed) using Mothur software and subsequently clustered at a 97% sequence identity using cd-hit to generate OTUs. The OTUs of the sequences were used to construct a sample-OTU count matrix. The samples were clustered at the genus and OTU levels using the sample-genus and sample-OTU count matrices, respectively. For each clustering, Morisita-Horn dissimilarity was used to construct a sample distance matrix from the initial count matrix, and the distance matrix was subsequently used for hierarchical clustering analysis using Ward’s minimum variance method. The Wilcoxon rank sum test was used to identify OTUs with differential abundances in the different sample groups.

For *Lactobacillus* isolation, fresh stool samples (100 mg) were collected, diluted in 2 ml of PBS solution and cultured on Rogosa SL selective medium (Sigma-Aldrich) for *Lactobacillus* enumeration, and the colonies were then identified and purified using 16S ribosomal DNA sequence analyses for the speciation of colonial genotypes. The lactobacilli were cultured in deMan, Rogosa, Sharpe (MRS; 3 M Health Care, St. Paul, MN, USA) media and grown on MRS agar containing 10% sucrose. Anaerobic conditions were generated with AnaeroPack-Anaero sachets (Mitsubishi Gas Chemical, Japan) in an airtight jar.

For the assessment of *Lactobacillus* IAA production *in vitro*, the *Lactobacilli* were propagated routinely for 24 h at 37 °C in MRS broth medium. Monoclonal *Lactobacillus* was newly propagated in MRS broth with or without 3 mM tryptophan to induce tryptophan catabolism. The supernatants were collected at the indicated time points, and IAA was analyzed.

### *IaaM* gene deletion in *Lactobacillus*

For *Lactobacillus iaaM* gene deletion, the upstream and downstream fragments of the *iaaM* gene from Lactobacillus were first amplified. The purified upstream and downstream homologous fragments were inserted into the Xoh I, Pem I, Sac I, and Bgl II digestion sites of the pNZ5319 plasmid. Receptive *Lactobacillus* cells were prepared, and the recombinant pNZ5319 plasmid was electrotransferred into the receptive *Lactobacillus* cells with the electrotransfer parameters of 1.7 kV (2 mm electrode cup), 200 Ω resistance and 25 μF capacitance. Single colonies of chloramphenicol-resistant *Lactobacillus* cells were selected, and the iaaM-up-F/R, iaaM-down-F/R, and CM-F/R primers were used to validate the strains with single exchanges. The single-exchange strains were cultured for 3 generations per day at 30 °C, and the suspensions were acquired at 40 generations for double-exchange strain screening. Colonies that grew normally on chloramphenicol-resistant plates but not on solid erythromycin-resistant plates were selected. The screened double exchangers were verified using iaaM-F/R PCR. Double-exchange–positive bacteria were prepared as receptor cells and electrotransfected together with the pNZTs-Cre plasmid for 3 generations per day for approximately 10 generations, eliminating the chloramphenicol resistance gene from the genome; the resultant plasmid was verified using CM-F/R PCR. The heat-sensitive plasmid pNZTs-Cre was eliminated by incubation at 42 °C for 3–5 h.

### LC-MS (liquid chromatography–mass spectrometry)/MS

After thawing at room temperature, all samples were extracted with methanol, and an internal standard (2.9 mg/ml, DL-O-chlorophenylalanine) was added. The samples were vortexed for 30 s and centrifuged at 12,000 rpm for 15 min at 4 °C. The samples were purified on a Waters ACQUITY UPLC HSS T3 column, and analyzed by ACQUITYTM UPLC-QTOF. The data were extracted and preprocessed with Masslynx 4.1 software (Waters) and then normalized and edited into a two-dimensional data matrix by Excel 2010 software; the matrix included the retention time (RT), mass, observations (samples), and peak intensity. After editing, the data were analyzed using SIMCA-P 13.0 software (Umetrics AB, Umea, Sweden).

### Cell isolation and flow cytometry

Cell isolation and flow cytometry were performed in accordance with a previously reported protocol [68]. Briefly, for the staining of immune cells in adipose tissues, adipose tissues were first cut into smaller pieces and then digested in digestion buffer (1 mg/ml collagenase I, Sigma-Aldrich) for 35 min. The digested tissues were then filtered through a 40-mm filter. Single-cell suspensions of mouse splenic PPs were prepared by mashing in a cell strainer (70 mm). For the staining of LP lymphocytes, gut tissues were isolated and cleaned by shaking in ice-cold PBS before being cut into 1-cm pieces. The epithelial cells were removed by incubating the tissue in HBSS with 2 mM EDTA for 30 min at 37 °C while shaking. LP cells were isolated by incubating the tissues in digestion buffer (DMEM, 5% fetal bovine serum, 1 mg/ml collagenase IV (Sigma-Aldrich) and DNase I (Sigma-Aldrich) for 40 min. The digested tissues were then filtered through a 40-mm filter. Cells were resuspended in 10 ml of the 40% fraction of a 40:80 Percoll gradient and overlaid onto 5 ml of the 80% fraction in a 15-ml Falcon tube. Percoll gradient separation was performed by centrifugation for 20 min at 1800 rpm at room temperature. LP cells were collected at the interphase of the Percoll gradient, washed and resuspended in medium, and then stained and analyzed by flow cytometry. Dead cells were eliminated through 7-AAD staining.

For the analysis of different immune cell populations, the cells were washed with staining buffer containing 2% FBS, 1 mM EDTA and 0.09% NaN3, and surface staining was performed with APC-, FITC-, PercP-, BV 605- or PE-labeled antibodies; the results were analyzed using FACScan flow cytometry. For intracellular staining, the cells were cultured and stimulated with 50 ng/ml phorbol 12-myristate 13-acetate (PMA, Sigma) and 1 μg/ml ionomycin (Sigma) in the presence of GolgiStop (10 ng/ml, BD Biosciences). After incubation for 6 h, the cells were washed with PBS, fixed in Cytofix/Cytoperm, permeabilized with Perm/Wash buffer (BD Biosciences), and stained with FITC-, PE-, APC- APC/Cy7-, PerCP/Cy5.5- or PE/Cy7-conjugated antibodies. The dead cells were eliminated through 7-AAD staining. For the absolute quantification of cell counts, the mouse tissues were weighed, and single-cell suspensions were prepared for flow cytometry. The total number of cells in per gram of tissue was counted and then multiplied by the proportion of positive cells to obtain the absolute cell number.

### CHIP-seq and CHIP-PCR

Chromatin immunoprecipitation (ChIP)-PCR was performed using the EZ-CHIP™ Chromatin Immunoprecipitation Kit (Millipore) according to our previously reported method [69]. Briefly, cells were washed with ice-cold PBS (containing 1% PMSF) and immediately resuspended in SDS lysis buffer (containing 1% PMSF). Cell lysates were sonicated for 40 cycles of 30 s on and 30 s off in 10-cycle increments using a Biorupter (Diadenode) on ice. After pelleting the debris, protein G agarose was added for 1 h at 4 °C with rotation for preclearing. For IP, the precleared cell lysate was incubated with the indicated antibodies overnight while rotating at 4 °C, and protein G agarose was added for the final 2 h of incubation. The beads were washed with low-salt, high-salt, and LiCl wash buffer, and chromatin immunocomplexes were eluted by incubation with the elution buffer at room temperature for 15 min. Reverse crosslinks of protein/DNA complexes to free DNA were induced by the addition of 5 M NaCl and incubation at 65 °C overnight. ChIP sequencing and qPCR analyses were performed after the treatment of purified DNA with RNase (30 min, 37 °C) and proteinase K (2 h, 55 °C) and after crosslink reversal.

### H & E staining, immunostaining, immunoprecipitation, immunoblot, PCR, qPCR, and ELISA

Hematoxylin/eosin (H&E) staining, immunostaining, IP, immunoblot, PCR, qPCR, and ELISA analyses were performed according to our previously reported methods [70].

### Statistical analyses

Student’s *t* test, one-way ANOVA with Bonferroni’s multiple comparison test, and the Mann–Whitney U test were used to determine significances. Correlations were assessed with the Spearman nonparametric rank test. A 95% confidence interval was considered significant and was defined as *p* < 0.05 (* *p* < 0.05, ** *p* < 0.01, *** *p* < 0.001).

## Supplementary Information

**Additional file 1: Figure S1**. Reg4 does not affect body weights under normal diet. **Figure S2**. Fluoresence minus one (FMO) controls for the flow cytometry plots of Fig. 2a (a) and b (b). **Figure S3**. Phenotypes of IL-35^+^ B cells in the adipose tissues of *huREG4*^*IECtg*^ mice. Flow cytometry of CD45^+^, CD19^+^, p35^+^Ebi3^+^ cells and the surface markers (IgD, IgM, IL-10, CD1d, CD5, CD11b, CD21/35, CD23, CD25, CD69, CD72, CD138, CD40 and CD86) of p35^+^Ebi3^+^ cells. Gray line, isotype negative controls (NC); Red line, surface markers in p35^+^Ebi3^+^B cells of WT mice; Blue line, surface markers in p35^+^Ebi3^+^ B cells of *huREG4*^*IECtg*^ mice (HuR4). **Figure S4**. Reg 4 promotes accumulation of IL-35^+^ cells in colonic lamina propria (LP) tissues, spleen and payer patch. **Figure S5**. The proportion of gut bacteria. **Figure S6**. Generation of CD19^+^p35^+^Ebi3^+^ or CD4^+^p35^+^Ebi3^+^ cells depends on gut microbiota. **Figure S7**. IAA plus LPS does not induce IL-35+CD4+ cells *in vitro*. **Figure S8**. IAA plus LPS promotes the binding of PXR with P65 and RXR. **Figure S9**. IAA with LPS mediated CD19+p35+Ebi3+ cells depends on PXR transcription factor. **Figure S10**. Dominant lactobacillus with LPS induces generation of CD19^+^p35^+^ or CD19^+^p35^+^Ebi3^+^ cells. **Figure S11**. CD45.1 cells in the adipose tissues of mice transplanted B cells. **Figure S12**. IL-35 promotes resistance to HFD induced obesity. Glucose tolerance (upper) and insulin sensitivity (below) of WT (WT1), *Reg4* KO(R4KO) and *huREG4*^*IECtg*^(HuR4) mice and WT2 which were fed by HFD for 10 weeks, and then transplanted using IL-35^+^ B cells with (siIL-35) or without siRNA treatment. Data are from three independent experiments. Analysis of variance test. *P < 0.05, **P < 0.01, and ***P < 0.001*.* Other cells, isolated B cells from spleen cells. **Figure S13**. F4/80^+^CD11C^+^, F4/80^+^CD206^+^, IFNγ^+^CD4^+^ and Foxp3^+^CD4^+^ cells in fat pad of different treated mice. **Table S1**. Reagents used in this study.

## Data Availability

RAW16S rRNA gene sequence data can be found at BioProject under accession number PRJNA695415 (http://www.ncbi.nlm.nih.gov/bioproject/695415).
