# Supplementary material for: Gut microbiota–derived metabolite 3-idoleacetic acid together with LPS induces IL-35+ B cell generation
Source: Microbiome. 2022 Jan 24;10:13. doi: 10.1186/s40168-021-01205-8 (PMC8785567; doi:10.1186/s40168-021-01205-8)
Supplement: Supplementary file 2 — Additional file 1: Figure S1. Reg4 does not affect body weights under normal diet. Figure S2. Fluoresence minus one (FMO) controls for the flow cytometry plots of Fig. 2a (a) and b (b). Figure S3. Phenotypes of IL-35+ B cells in the adipose tissues of huREG4IECtg mice. Flow cytometry of CD45+, CD19+, p35+Ebi3+ cells and the surface markers (IgD, IgM, IL-10, CD1d, CD5, CD11b, CD21/35, CD23, CD25, CD69, CD72, CD138, CD40 and CD86) of p35+Ebi3+ cells. Gray line, isotype negative controls (NC); Red line, surface markers in p35+Ebi3+B cells of WT mice; Blue line, surface markers in p35+Ebi3+ B cells of huREG4IECtg mice (HuR4). Figure S4. Reg 4 promotes accumulation of IL-35+ cells in colonic lamina propria (LP) tissues, spleen and payer patch. Figure S5. The proportion of gut bacteria. Figure S6. Generation of CD19+p35+Ebi3+ or CD4+p35+Ebi3+ cells depends on gut microbiota. Figure S7. IAA plus LPS does not induce IL-35+CD4+ cells in vitro. Figure S8. IAA plus LPS promotes the binding of PXR with P65 and RXR. Figure S9. IAA with LPS mediated CD19+p35+Ebi3+ cells depends on PXR transcription factor. Figure S10. Dominant lactobacillus with LPS induces generation of CD19+p35+ or CD19+p35+Ebi3+ cells. Figure S11. CD45.1 cells in the adipose tissues of mice transplanted B cells. Figure S12. IL-35 promotes resistance to HFD induced obesity. Glucose tolerance (upper) and insulin sensitivity (below) of WT (WT1), Reg4 KO(R4KO) and huREG4IECtg(HuR4) mice and WT2 which were fed by HFD for 10 weeks, and then transplanted using IL-35+ B cells with (siIL-35) or without siRNA treatment. Data are from three independent experiments. Analysis of variance test. *P < 0.05, **P < 0.01, and ***P < 0.001. Other cells, isolated B cells from spleen cells. Figure S13. F4/80+CD11C+, F4/80+CD206+, IFNγ+CD4+ and Foxp3+CD4+ cells in fat pad of different treated mice. Table S1. Reagents used in this study. [file 40168_2021_1205_MOESM1_ESM.docx]

Supplementary information

**Gut microbiota-derived metabolite 3-idoleacetic acid together with LPS induces IL-35^+^ B cell generation**

Xiaomin Su^3^, Minying Zhang^3^, Houbao Qi^1.3^, Yunhuan Gao^3^, Yazheng Yang^3^, Huan Yun^3^, Qianjing Zhang^3^, Xiaorong Yang^3^, Yuan Zhang ^3^, Jiangshan He ^3^, Yaqi Fan^3^, Yuxue Wang^3^, Pei Guo^3^, Chunze Zhang^4^ & Rongcun Yang^1, 2, 3^


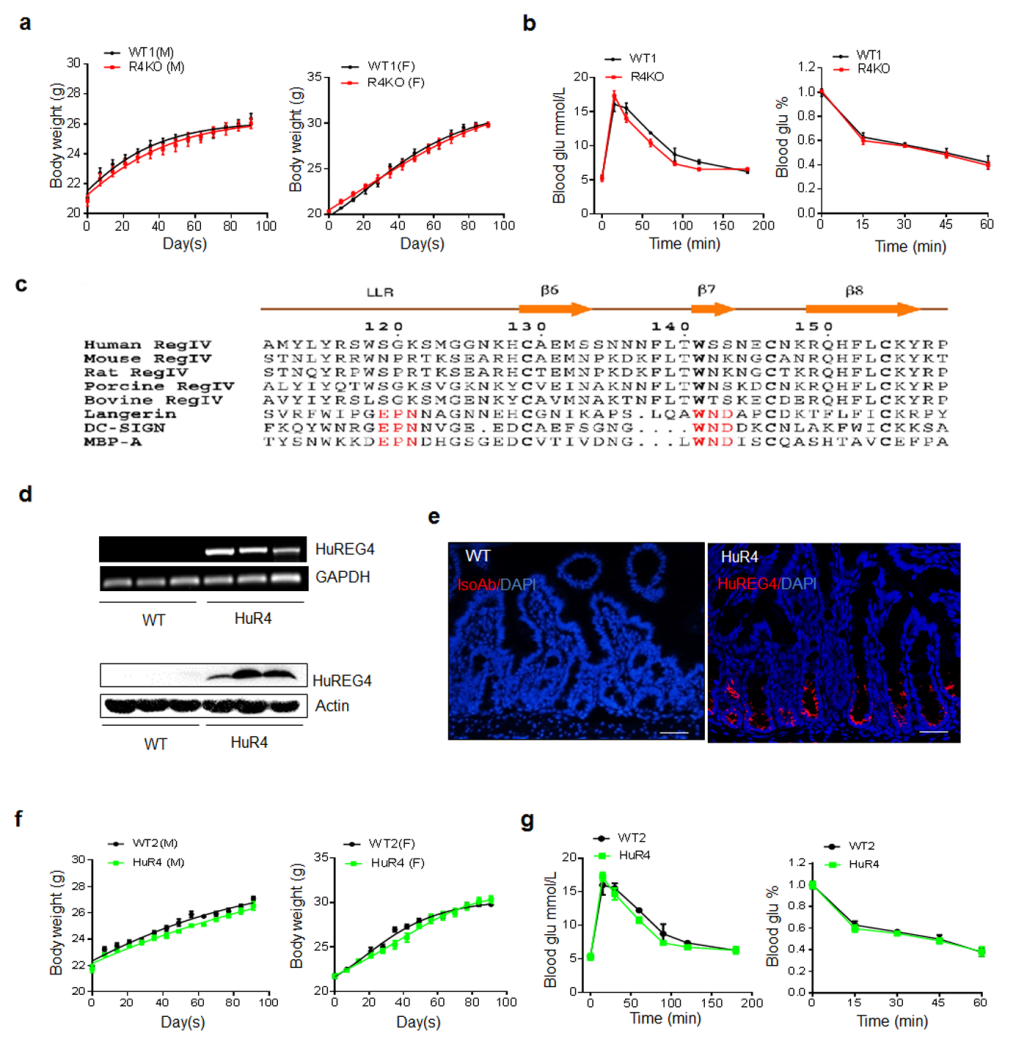


**Figure S1. Reg4 does not affect body weights under normal diet.**

**a** Body weights of male (left) or female (right) WT(WT1) and *Reg4* KO mice (R4KO) fed normal diet (n=14).

**b** Glucose tolerance and insulin sensitivity of WT (WT1) and *Reg4* KO mice (R4KO) fed normal diet (n=6).

**c** Higher conservation of Reg4 among different animal species.

**d** PCR and Western bot of human REG4 in *huREG4^IECtg^* mice(HuR4). WT1, WT2 and WT3, different wild type mice; HuR41, 2 and 3, different *huREG4^IECtg^* mice.

**e** Immunostaining of human REG4 in WT and *huREG4^IECtg^* mice (HuR4).IsoAb, isotypic control. Scale bars in **e**=40 µm.

**f** Body weights of male (left) or female (right) WT (WT2) and *huREG4^IECtg^* mice (HuR4) fed normal diet (n=14).

**g** Glucose tolerance and insulin sensitivity of WT (WT2) and *huREG4^IECtg^* mice (HuR4) fed normal diet (n=6).

Analysis of variance test in **a**, **b**, **f** and **g**，no difference.


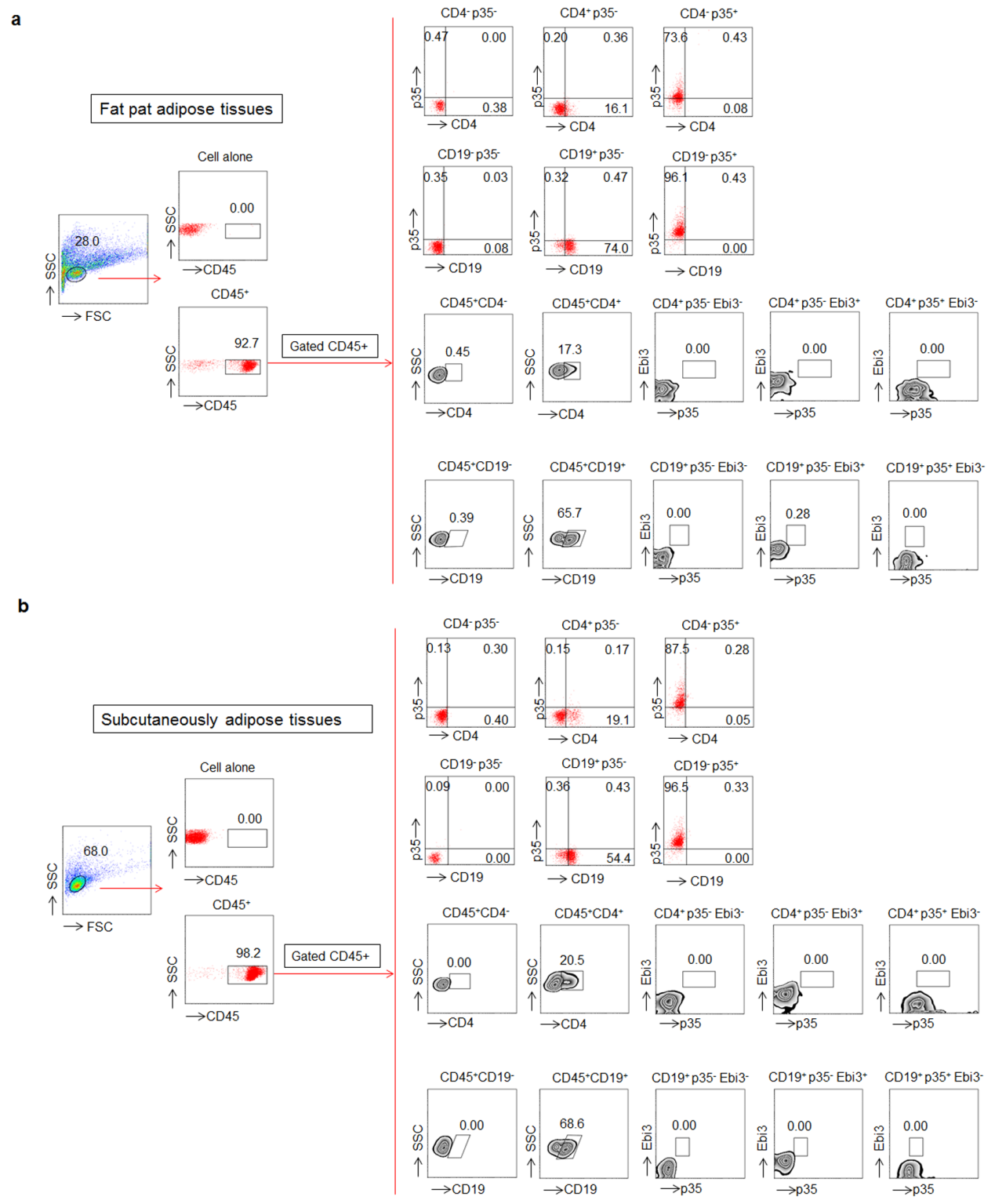


**Figure S2. Fluoresence minus one (FMO) controls for the flow cytometry plots of Figure 2a (a) and 2b (b).**


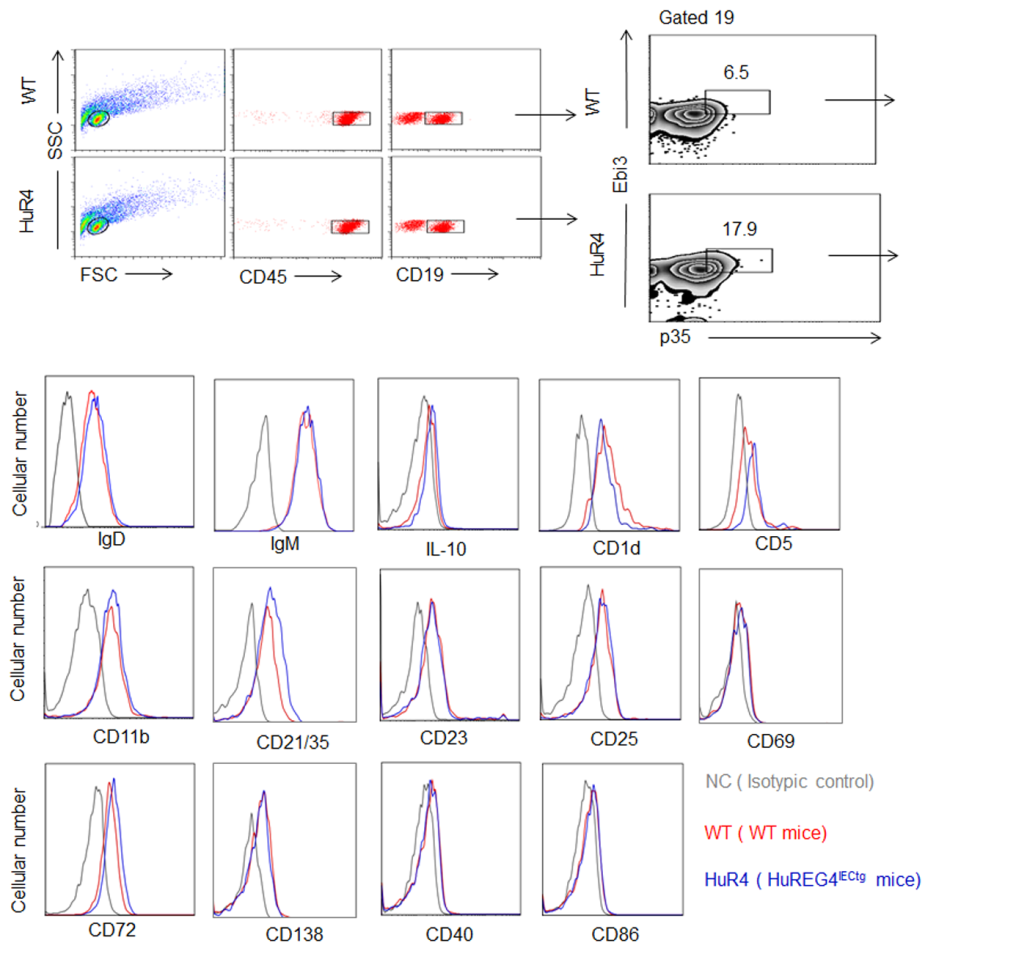


**Figure S3. Phenotypes of IL-35^+^ B cells in the adipose tissues of *huREG4^IECtg^* mice.** Flow cytometry of CD45^+^, CD19^+^, p35^+^Ebi3^+^ cells and the surface markers (IgD, IgM, IL-10, CD1d, CD5, CD11b, CD21/35, CD23, CD25, CD69, CD72, CD138, CD40 and CD86) of p35^+^Ebi3^+^ cells.

Gray line, isotype negative controls (NC); Red line, surface markers in p35^+^Ebi3^+^B cells of WT mice; Blue line, surface markers in p35^+^Ebi3^+^ B cells of *huREG4^IECtg^* mice (HuR4).


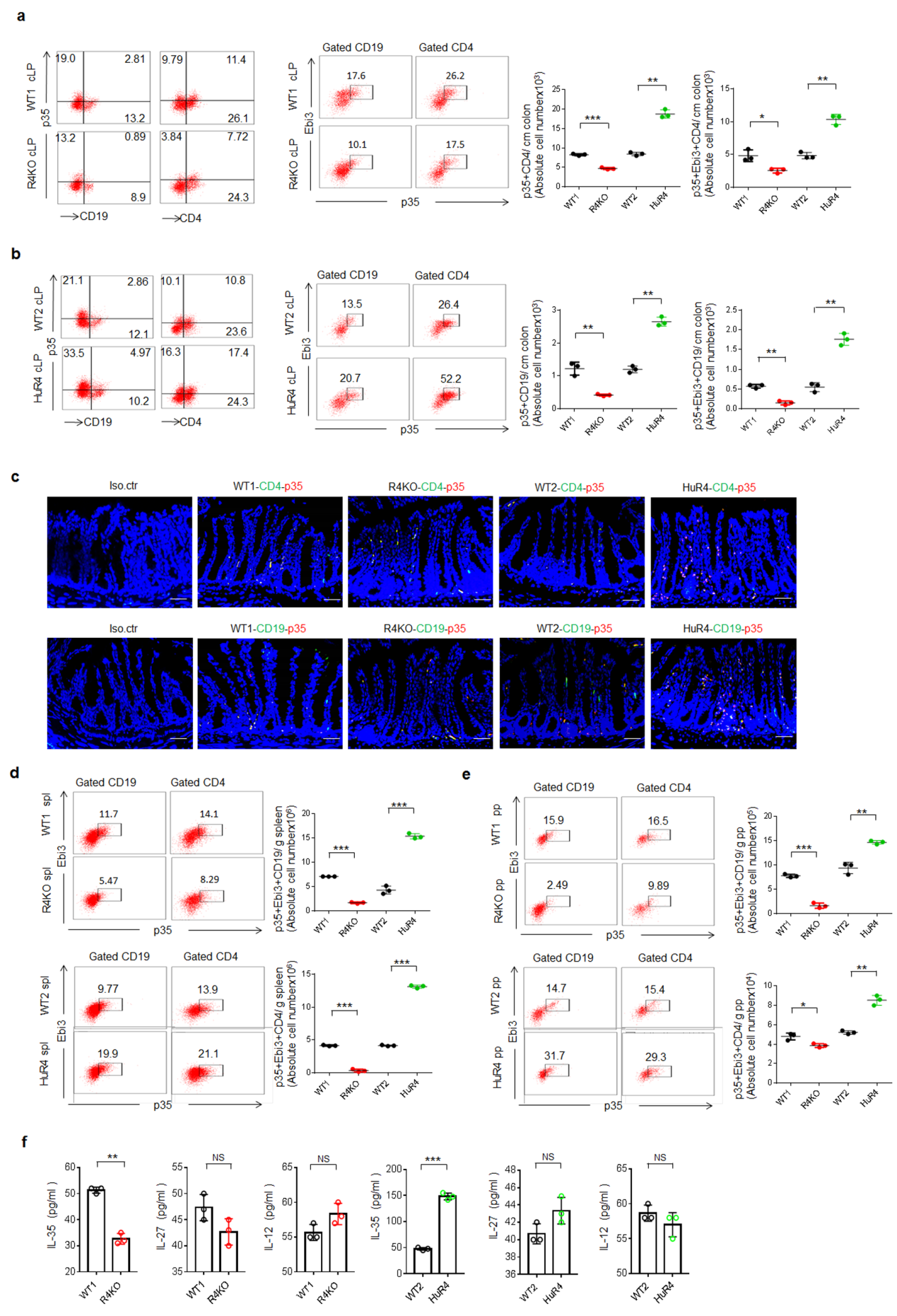


**Figure S4. Reg 4 promotes accumulation of IL-35^+^ cells in colonic lamina propria (LP) tissues, spleen and payer patch.**

**a** and **b** Flow cytometry of p35^+^CD19^+^, p35^+^CD4^+^, p35^+^Ebi3^+^CD19^+^ and p35^+^Ebi3^+^CD4^+^ cells in the colonic LP (cLP) of *Reg4* KO (R4KO) and control WT (WT1) (**a**) or *huREG4^IECtg^* (HuR4) and their littermate control mice (WT2) (**b**) fed HFD for three months.

**c** Immunostaining of p35^+^CD19^+^ and p35^+^CD4^+^ in the colon tissue of *Reg4* KO (R4KO) and control WT(WT1) or *huREG4^IECtg^* (HuR4) and their littermate control mice(WT2) fed HFD for three months

**d** and **e** Flow cytometry of p35^+^Ebi3^+^CD19^+^ and p35^+^Ebi3^+^CD4^+^ cells in spleen (**d**) the payer patch (PP) (**e**) of *Reg4* KO (R4KO) and control WT (WT1)or *huREG4^IECtg^* (HuR4) and their littermate control mice (WT2) fed HFD for three months.

**f** ELISA of IL-35, IL-27 and IL-12 in the colon tissues of *Reg4* KO (R4KO) and control WT (WT1) or *huREG4^IECtg^* (HuR4) and their littermate control mice (WT2) fed HFD for three months. Gut tissues was milled in liquid nitrogen and then lysed with non-denatured tissue lysis solution for ELISA. Mixed sample from 12 mice.

Data in **a**, **b**, **d** and **e** are from three independent experiments. Student’s *t*-test in all panels, mean ±SD; **P* < 0.05, ***P* < 0.01, and ****P* < 0.001; NS, No significance*.*


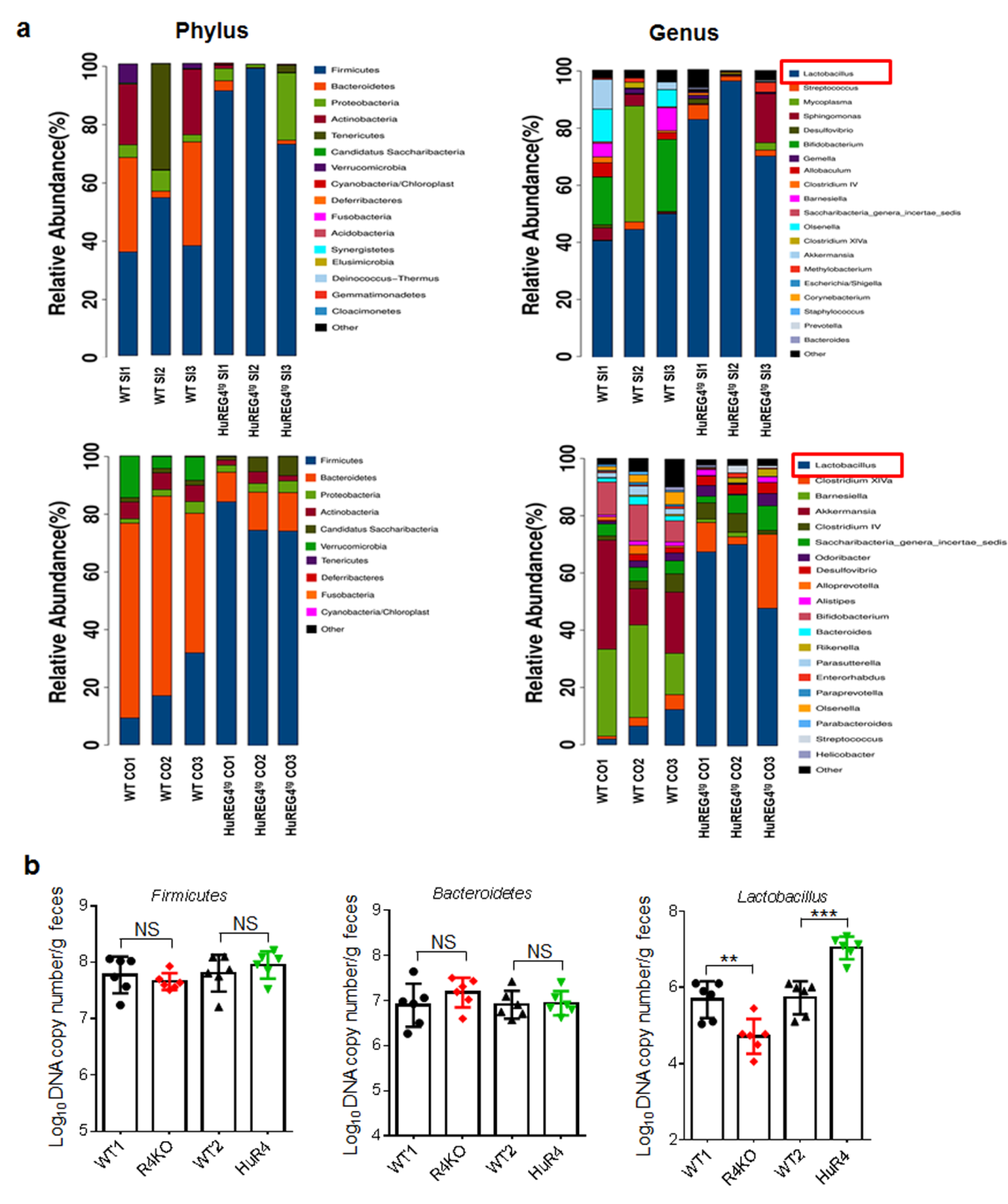


**Figure S5. The proportion of gut bacteria.**

**a** The proportion of gut bacteria after 16S rRNA-seq of gut microbiota from pooled ileum content (S.I) and colon content (Co) samples of WT and *huREG4^IECtg^* (HuREG4^tg^) mice (Three independent experiments, 5 mice (7-8 weeks old, male)/ time).

**b** Q-PCR of *Firmicutes*, *bacteroidetes* and *lactobacillus* bacteria in colon contents of WT1 and Reg4KO (R4KO), WT2 and huREG4IENtg (HuR4) mice fed normal chow (n=6). Standard curves were prepared from serial dilustion of *Firmicutes*, *bacteroidetes* and *lactobacillus* 16S rRNA.

Student’s *t*-test in **b**, mean ±SD. **P* < 0.05, ***P* < 0.01, and ****P* < 0.001; NS, No significance*.*


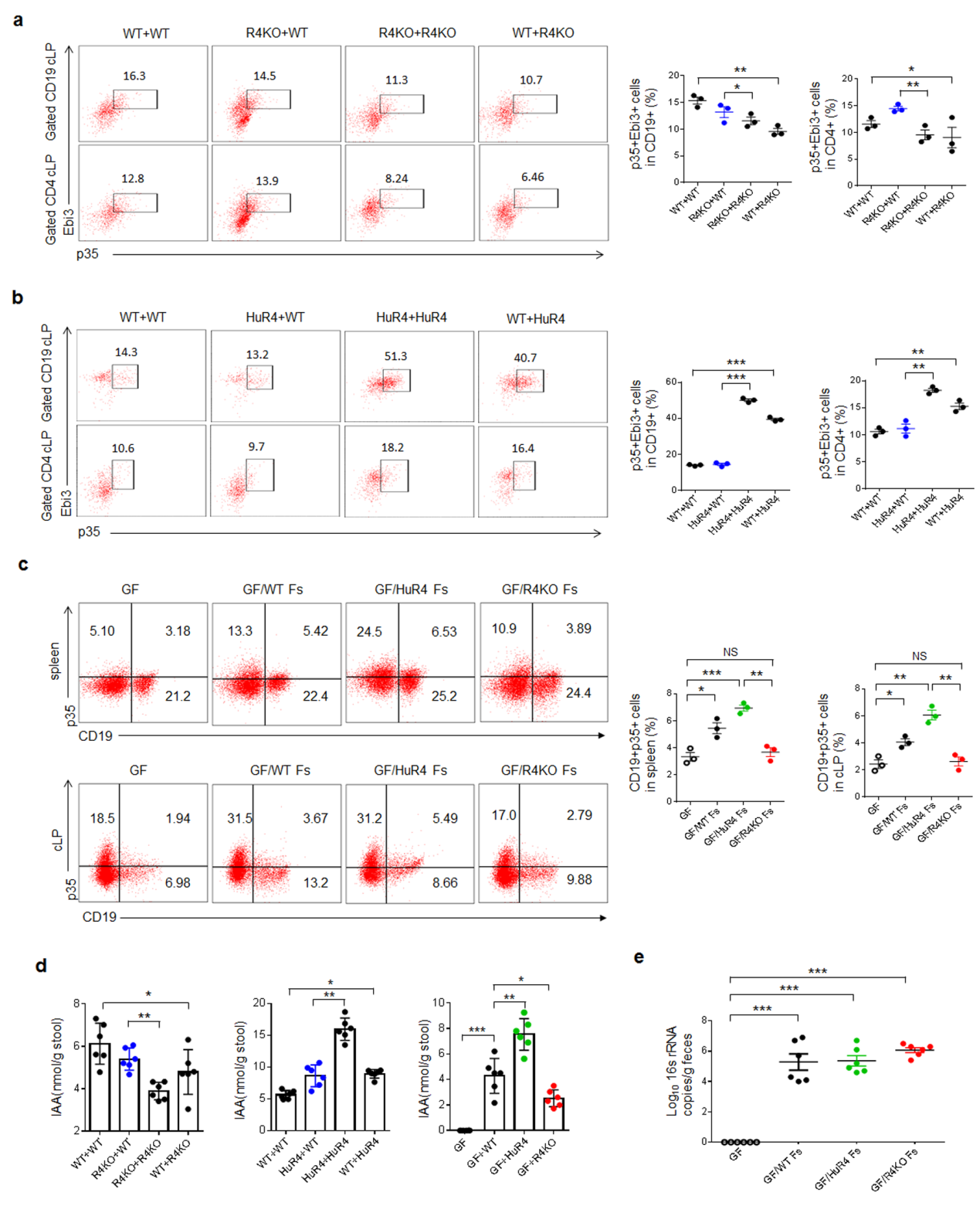


**Figure S6. Generation of CD19^+^p35^+^Ebi3^+^ or CD4^+^p35^+^Ebi3^+^ cells depends on gut microbiota.**

**a** and **b** Flow cytometry of CD19^+^p35^+^Ebi3^+^ or CD4^+^p35^+^Ebi3^+^ cells in the colonic LP (cLP) of different mice.

**c** Flow cytometry of CD19^+^p35^+^ or CD4^+^p35^+^ cells in the spleen or colon LP (cLP) of different mice.

**d** IAA levels in fresh feces of different mice.

**e** 16 S rRNA analyses of total bacteria in the fresh feces of different mice.

WT mice infused by WT mouse contents (WT+WT), WT mice infused by *Reg4* KO (WT+R4KO) or *huREG4^IECtg^* (WT+HuR4) mouse colon contents, *Reg4* KO mice infused by WT (R4KO+WT) or *Reg4* KO (R4KO+R4KO) mouse colon contents, -*huREG4^IECtg^* mice infused by WT (HuR4+WT) or *huREG4^IECtg^*（HuR4+HuR4）mouse colon contents in **a** and **b**;

GF mice infused by WT (GF/WT Fs), *huREG4^IECtg^* (GF/HuR4 Fs）or Reg4 KO (GF/R4KO Fs) colon contents in **c**, **d**, and **e**.

Data in **a**，**b** and **c** were from three independent experiments. Data in **d** and **e** were from one representative.

Student’s *t*-test in per panel, mean ±SD. **P* < 0.05, ***P* < 0.01, and ****P* < 0.001; NS, No significance*.*


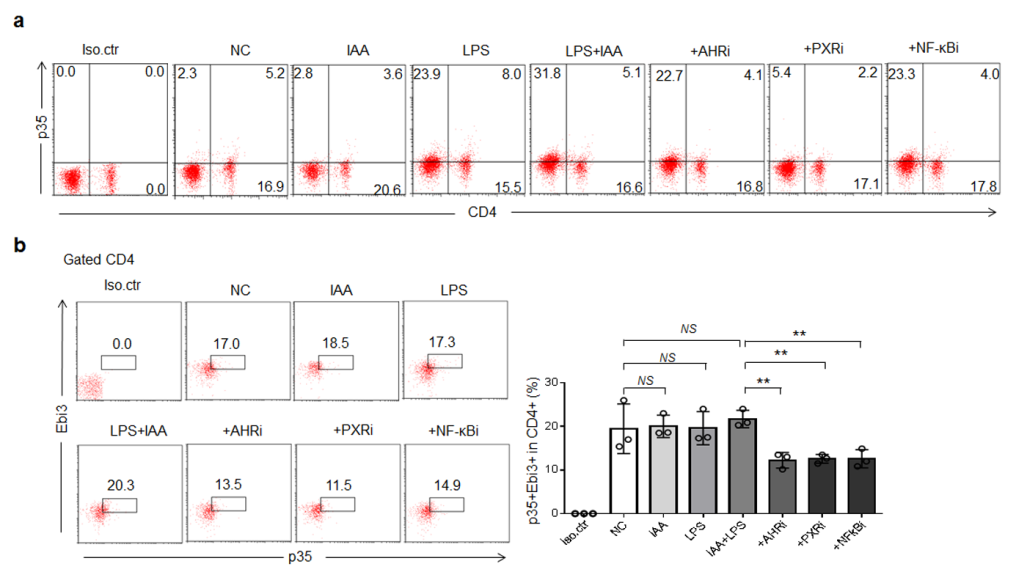


**Figure S7. IAA plus LPS does not induce IL-35+CD4+ cells *in vitro*.**

**a** Flow cytometry of p35^+^CD4^+^ cells in the spleen cells after exposed to IAA, LPS, LPS+IAA, LPS+IAA+AHR inhibitor (+AHRi), LPS+IAA+PXR inhibitor (+PXRi), LPS+IAA+NF-κB inhibitor (+NF-κBi) for 24 hrs.

**b** Flow cytometry of p35^+^Ebi3^+^CD4^+^ cells in the spleen cells after exposed to IAA, LPS, LPS+IAA，LPS+IAA+AHR inhibitor (+AHRi), LPS+IAA+PXR inhibitor (+PXRi), LPS+IAA+NF-κB inhibitor (+NF-κBi) for 24 hrs.

Data were from three independent experiments.

Student’s *t*-test, mean ±SD. **P* < 0.05, ***P* < 0.01, and ****P* < 0.001; NS, No significance*.*


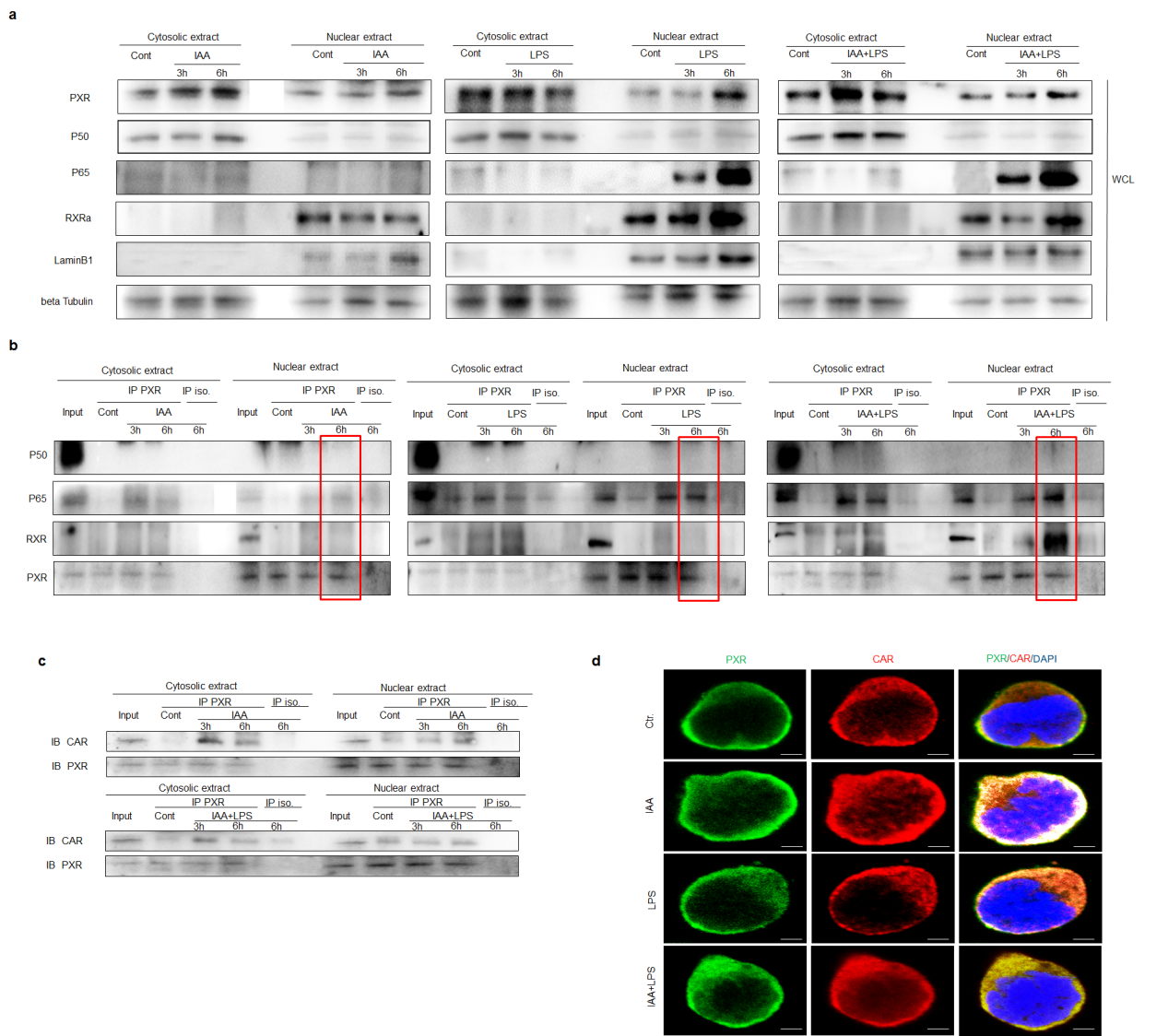


**Figure S8. IAA plus LPS promotes the binding of PXR with P65 and RXR.**

**a** Immunoblotting of PXR, P50, P65 and RXRa in cytoplasmic or nuclear extracts after exposed to IAA, LPS or IAA+LPS.

**b** Immunoblotting of P50, P65 and RXRa in the cytoplasmic or nuclear extracts from immunoprecipitation by anti-PXR antibody after exposed to IAA, LPS or IAA+LPS.

**c** Immunoblotting of CAR and PXR of cytosolic extracts and nuclear extracts after immunoprecipitation using anti-PXR in the WEHI231 B cells after exposed to IAA or IAA+LPS.

**d** Immunostaining of PXR and CAR in the WEHI231 B cells after exposed to IAA+LPS for 3hrs.


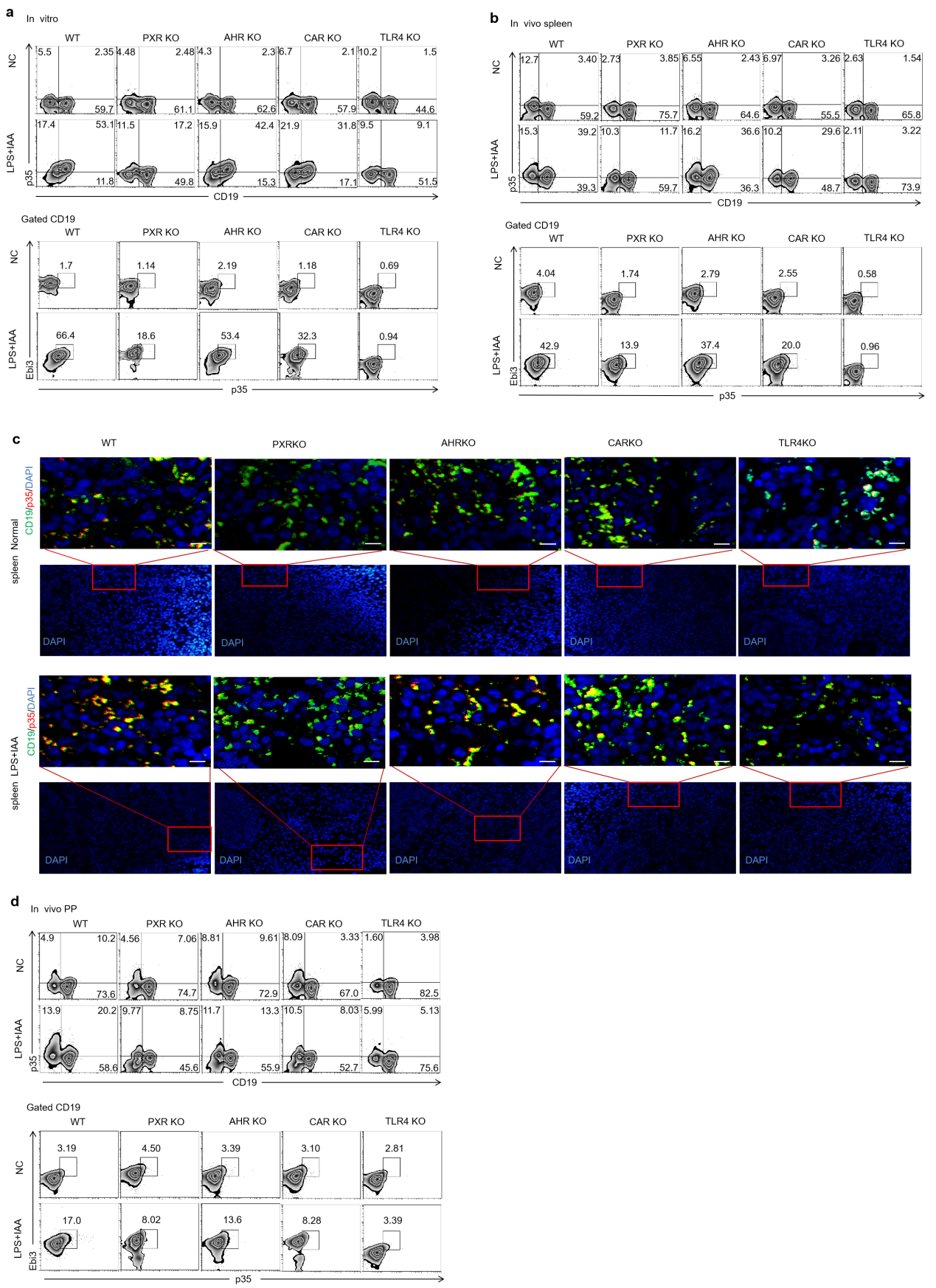


**Figure S9. IAA with LPS mediated CD19+p35+Ebi3+ cells depends on PXR transcription factor.**

**a** Flow cytometry of CD19^+^p35^+^, CD19^+^p35^+^Ebi3^+^ cells in the spleen cells after exposed to IAA+LPS *in vitro*.

**b** Flow cytometry of CD19^+^p35^+^, CD19^+^p35^+^Ebi3^+^ cells in the spleen after injecting IAA+LPS in vivo.

**c** Immunostaining of CD19^+^p35^+^ cells in the spleen with (IAA+LPS)or without (Normal) injecting IAA+LPS in vivo.

**d** Flow cytometry of CD19^+^p35^+^, CD19^+^p35^+^Ebi3^+^ cells in the PP after injecting IAA+LPS. Scale bars in **c** = 40 µm.

Data were from one representative.


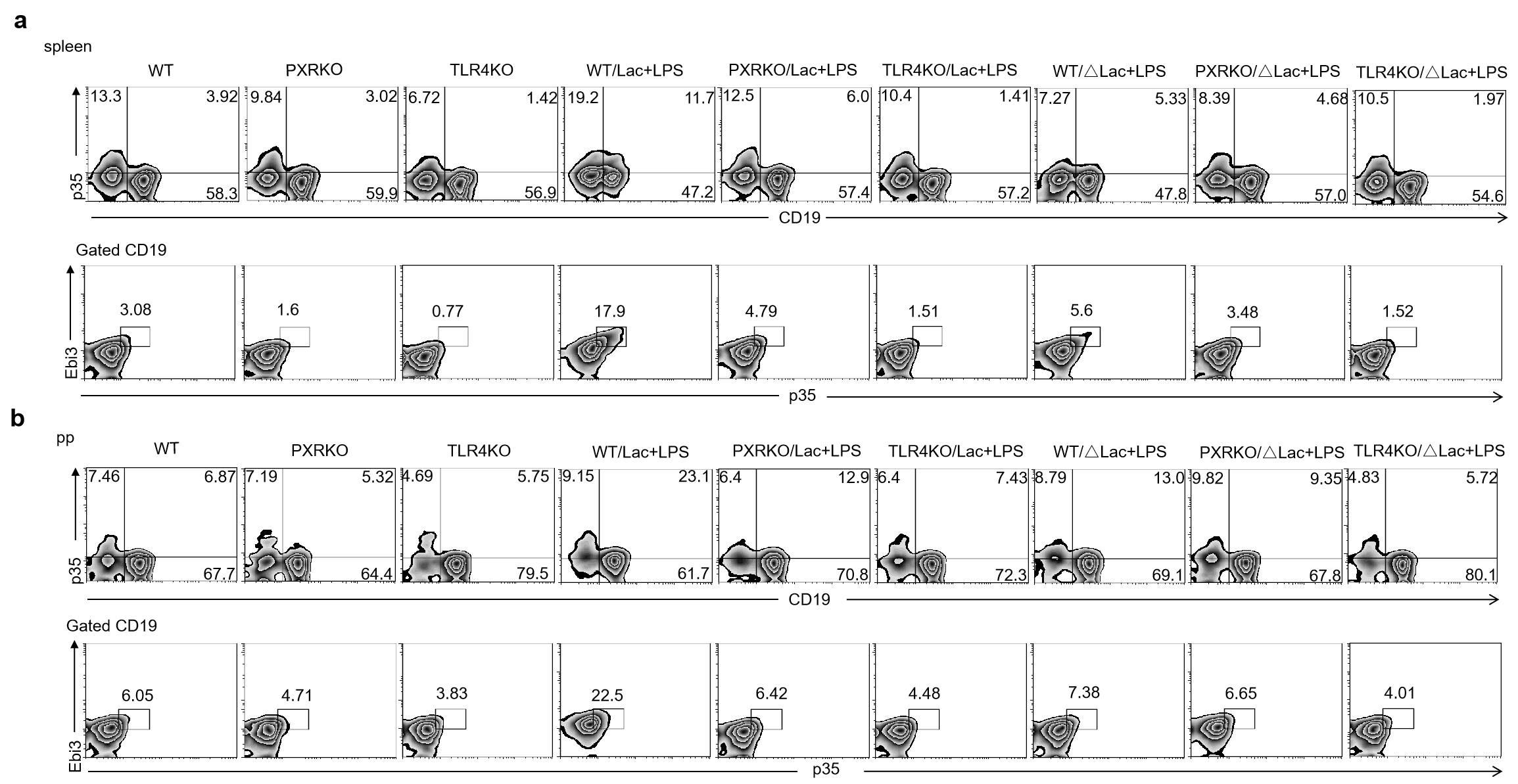


**Figure S10. Dominant lactobacillus with LPS induces generation of CD19^+^p35^+^ or CD19^+^p35^+^Ebi3^+^ cells.**

Flow cytometry of CD19^+^p35^+^or CD19^+^p35^+^Ebi3^+^ cells in the spleen (**a**) and PP (**b**) after infusing lactobacillus or mutated lactobacillus*^ΔiaaM^*. One representative.

**
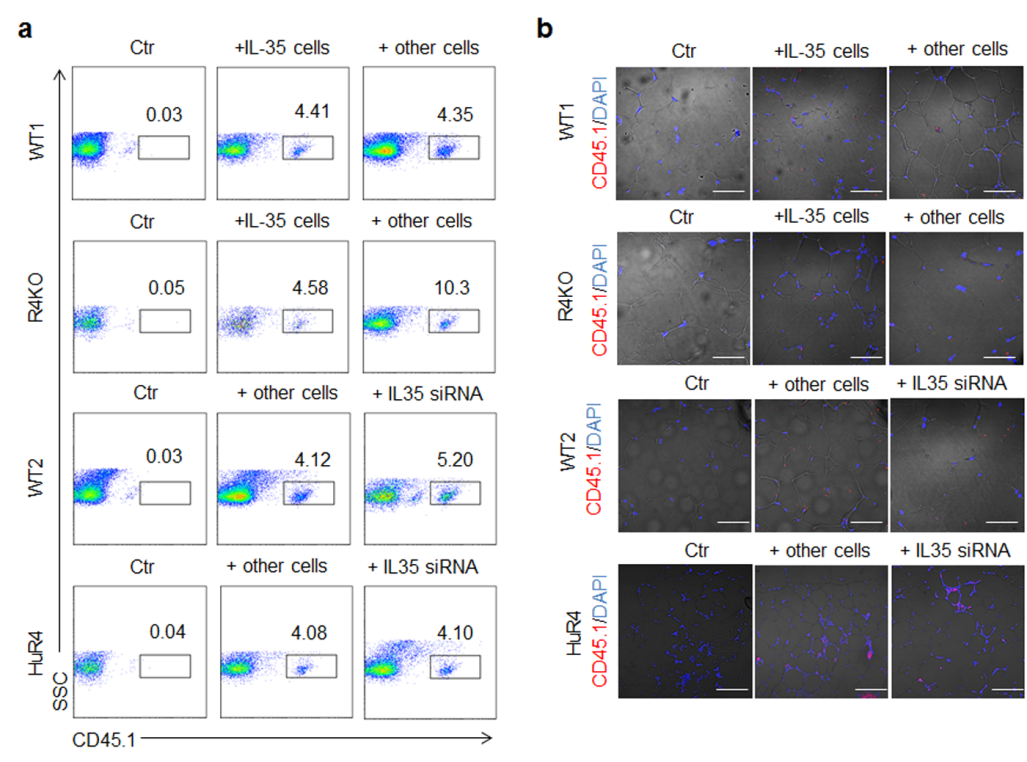
**

**Figure S11. CD45.1 cells in the adipose tissues of mice transplanted B cells.**

**a** Flow cytometry of CD45.1 cells in adipose tissues of different mice. Mice were injected CD45.1 IL-35+ B with (+IL-35 siRNA) or without siRNA treatment, and then were examined after 3 days.

**b** Immunostaining of CD45.1 cells in adipose tissues of different mice. Mice were injected CD45.1 IL-35+ B with (+IL-35 siRNA) or without siRNA treatment, and then were examined after 3 days.

Other cells, isolated B cells from spleen.


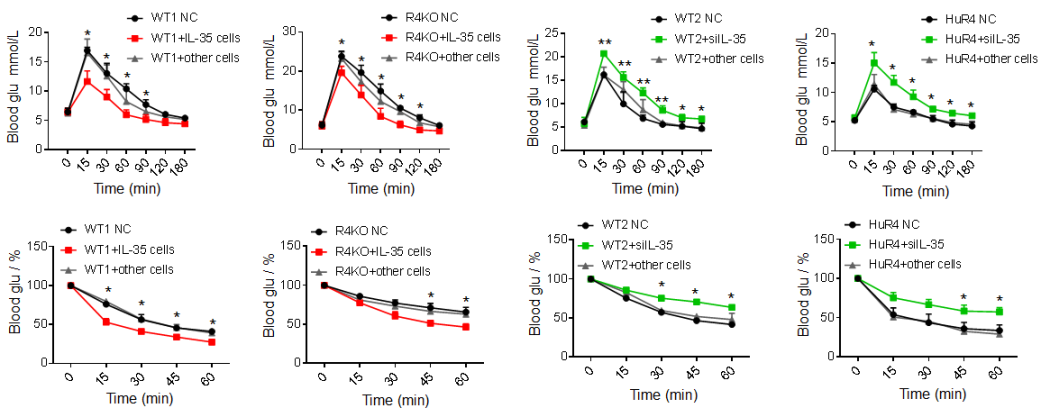


**Figure S12. IL-35 promotes resistance to HFD induced obesity.**

Glucose tolerance (upper) and insulin sensitivity(below) of WT (WT1), *Reg4* KO(R4KO) and *huREG4^IECtg^*(HuR4) mice and WT2 which were fed by HFD for 10 weeks, and then transplanted using IL-35^+^ B cells with (siIL-35) or without siRNA treatment. Data are from three independent experiments

Analysis of variance test. **P* < 0.05, ***P* < 0.01, and ****P* < 0.001*.* Other cells, isolated B cells from spleen cells.


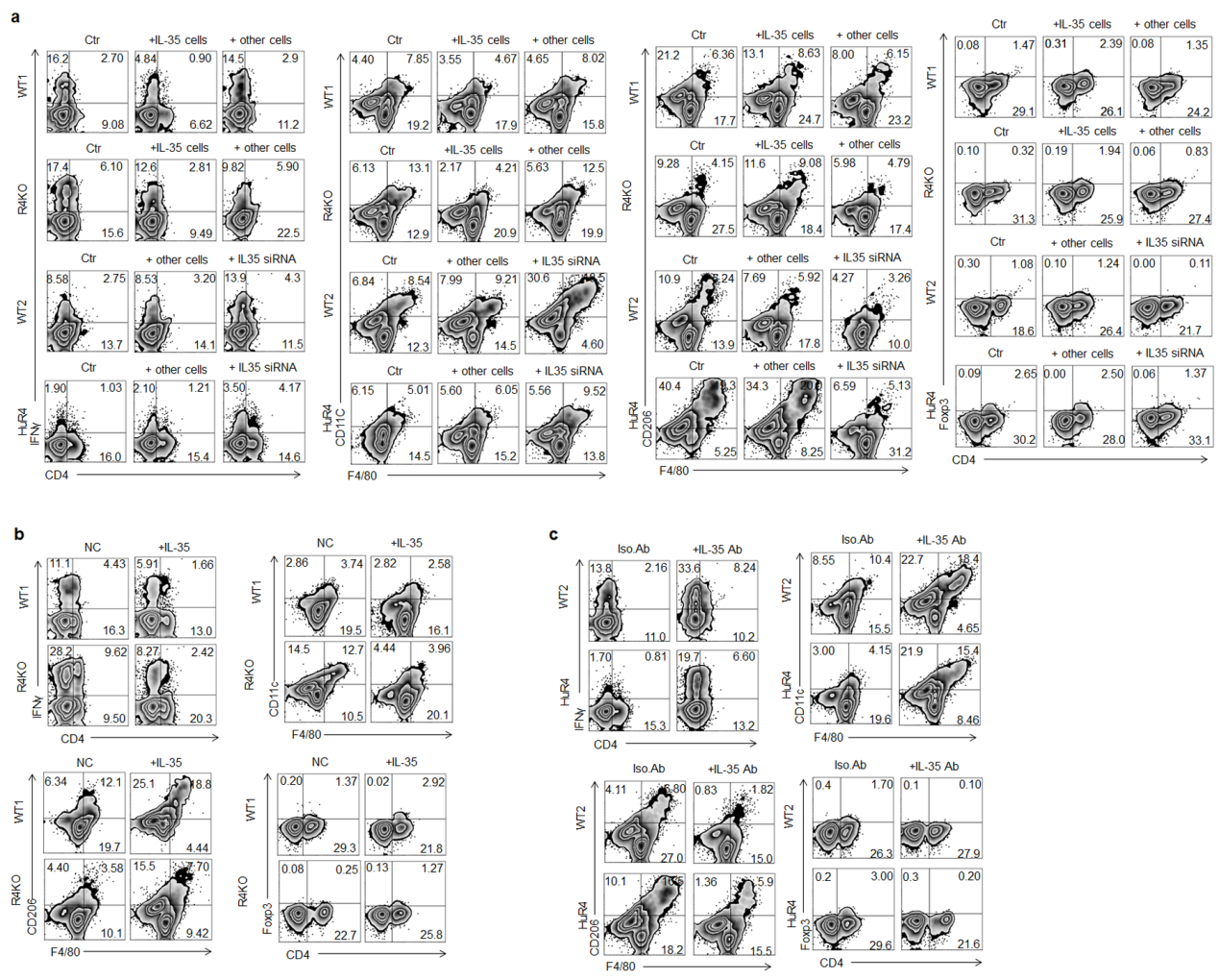


**Figure S13.** F4/80^+^CD11C^+^, F4/80^+^CD206^+^, IFNγ^+^CD4^+^ and Foxp3^+^CD4^+^ cells in fat pad of different treated mice.

**a** Flow cytometry of IFNγ^+^CD4^+^, F4/80^+^CD11C^+^, F4/80^+^CD206^+^ cells, and Foxp3^+^CD4^+^ in fat pad from WT1 and Reg4 KO (R4KO), WT2 and *HuREG4^IECtg^* mice(HuR4) transplanted using IL-35+ B cells with (IL-35siRNA) or without siRNA treatment. Other cells, isolated B cells from spleen.

**b** Flow cytometry of IFNγ^+^CD4^+^, F4/80^+^CD11C^+^, F4/80^+^CD206^+^, and Foxp3^+^CD4^+^ cells in fat pad from WT(WT1) and Reg4 KO mice (R4KO) with (IL-35) or without rIL-35 (NC).

**c** Flow cytometry of IFNγ^+^CD4^+^, F4/80^+^CD11C^+^, F4/80^+^CD206^+^, and Foxp3^+^CD4^+^ cells in fat pad from WT (WT2) and *HuREG4^IECtg^* mice (HuR4) with (IL-35Ab) or without anti-IL-35 blocking antibody (Iso Ab).

Data were from one representative.

**Table S1. Reagents used in this study.**

| REAGENT or RESOURCE | SOURCE | IDENTIFIER |
| --- | --- | --- |
| Antibodies for Western blot | | |
| β-Actin Antibody | Santa Cruz | Cat:sc-47778 RRID:AB_626632 |
| Anti-PXR Antibody | Cloud-clone | Cat:PAD950Mu01 |
| Anti-AHR Antibody | Proteintech | Cat:17840-1-AP RRID:AB_2226163 |
| Anti-P50 Antibody | Abcam | Cat:ab32360 RRID:AB_776748 |
| Anti-P65 Rabbit monoclonal Antibody | Abcam | Cat:ab32536 RRID:AB_776751 |
| Anti-RXRA Antibody | Abcam | Cat:ab125001 RRID:AB_10975632 |
| Anti-Lamin B1 Antibody | Abcam | Cat:ab16048 RRID:AB_443298 |
| Anti- beta Tubulin Antibody | Abcam | Cat:ab6046 RRID:AB_2210370 |
| Anti-CAR Antibody | Affinity | Cat:DF6725 RRID:AB_2838687 |
| Anti-PXR FITC Antibody | Cloud-clone | Cat:LAD950Mu81 |
| Anti-Ebi3 Antibody | Abcam | Cat:ab124694 RRID:AB_10972489 |
| Goat anti-mouse IgG (H+L) -HRP Antibody | ABclonal | Cat:AS003 RRID:AB_2769851 |
| Goat anti-rabbit IgG (H+L) -HRP Antibody | ABclonal | Cat:AS014 RRID:AB_2769854 |
| Goat anti-mouse IgG (L) -HRP Antibody | ABclonal | Cat:AS062 RRID:AB_2864056 |
| mouse anti-rabbit IgG (L) -HRP Antibody | ABclonal | Cat:AS061 RRID:AB_2864055 |
| Antibodies for flow cytometry analysis |  | |
| FITC anti-human CD19 Antibody | Biolegend | Cat: 363007 RRID:AB_2564170 |
| PE anti-human Ebi3 Antibody | Biolegend | Cat: 360903 RRID:AB_2562879 |
| APC anti-human P35 Antibody | Biolegend | Cat: 831603 RRID:AB_2686933 |
| PE anti-mouse IL-10 Antibody | Biolegend | Cat: 505007 RRID:AB_315361 |
| APC anti-mouse CD19 Antibody | Sungenebiotech | Cat: M10191-11c RRID:AB_511 |
| FITC anti-mouse CD19 Antibody | Biolegend | Cat: 152404 RRID:AB_2629813 |
| FITC anti-mouse CD4 Antibody | Biolegend | Cat: 100406 RRID:AB_312691 |
| PE anti-mouse CD8 Antibody | Biolegend | Cat: 100708 RRID:AB_312747 |
| PE anti-mouse/human p35 Antibody | R&D | Cat:IC2191P RRID:AB_1964620 |
| PerCP anti-mouse Ebi3 Antibody | R&D | Cat:IC18341C RRID:AB_2097431 |
| APC anti-mouse CD11c Antibody | Biolegend | Cat: 117310 RRID:AB_ 313779 |
| APC/Cy7 anti-mouse/human CD11b Antibody | Biolegend | Cat: 101226 RRID:AB_ 830642 |
| FITC anti-mouse F4/80 Antibody | Biolegend | Cat: 123108 RRID:AB_893502 |
| PE anti-mouse CD206 Antibody | Biolegend | Cat: 141706 RRID:AB_10895754 |
| PE anti-mouse IFNγ Antibody | Biolegend | Cat: 505808 RRID:AB_315402 |
| APC anti-mouse FoxP3 Antibody | Invitrogen | Cat: A27056 RRID:AB_2536115 |
| FITC anti-mouse IgD Antibody | BD Biosciences | Cat: 562022 RRID:AB_10894208 |
| APC anti-mouse CD45.1 Antibody | Biolegend | Cat: 110714 RRID:AB_313503 |
| APC anti-mouse CD45 Antibody | Biolegend | Cat: 103112 RRID:AB_312977 |
| FITC anti-mouse CD1d Antibody | Thermo Fisher Scientific | Cat: 11-0011-82 RRID:AB_464867 |
| PE anti-mouse CD11b Antibody | Sungenebioth | Cat: M10117-09D |
| APC anti-mouse CD21/35 Antibody | Biolegend | Cat: 123411 RRID:AB_940395 |
| APC anti-mouse CD23 Antibody | Biolegend | Cat: 101619 RRID:AB_2563438 |
| FITC anti-mouse CD40 Antibody | BD Biosciences | Cat: 553790 RRID:AB_395054 |
| FITC anti-mouse CD86 Antibody | BD Biosciences | Cat: 553691 RRID:AB_394993 |
| PE anti-mouse CD5 Antibody | BD Biosciences | Cat: 553022 RRID:AB_394560 |
| PE anti-mouse CD25 Antibody | BD Biosciences | Cat: 553866 RRID:AB_395101 |
| 8FITC anti-mouse CD72 Antibody | BD Biosciences | Cat: 550966 RRID:AB_393982 |
| BV510 anti-mouse CD69 Antibody | Biolegend | Cat: 104531 RRID:AB_2562326 |
| PE anti-mouse IgD Antibody | BD Biosciences | Cat: 553511 RRID:AB_394894 |
| PE anti-mouse IgM Antibody | BD Biosciences | Cat: 553521 RRID:AB_394902 |
| PE anti-mouse CD138 Antibody | Biolegend | Cat: 142503 RRID:AB_10915989 |
| FITC anti-mouse IgG1 Antibody | BD Biosciences | Cat: 553443 RRID:AB_394862 |
| Anti-LPS Antibody | Meridian Life Science | Cat:B65692G RRID:AB_152277 |
| Anti-WGA Antibody | Invitrogen | Cat:W21405 |
| Anti-goat IgG (H+L), (Alexa Fluor 488 Conjugate) Antibody | Bioss | Cat:bs-0294R RRID:AB_10855262 |
| Antibodies for immunostaining |  | |
| Anti-Reg4 Antibody | Santa Cruz Biotechnolgy | Cat:**s**c-48077 RRID:AB_2178709 |
| Anti-P35Antibody | Proteintech | Cat:55018-1-AP RRID:AB_10860266 |
| Anti-CD4 Antibody | eBioscience | Cat:11-0041-82 RRID:AB_464892 |
| Anti-CD19 Antibody | eBioscience | Cat:14-0194-82 RRID:AB_2637170 |
| Anti-P65 mouse monoclonal Antibody | Proteintech | Cat:66535-1-Ig RRID:AB_2881898 |
| Anti-PXR FITC Antibody | Cloud-clone | Cat:LAD950Mu81 |
| Anti-RXRA Antibody | Abcam | Cat:ab125001 RRID:AB_10975632 |
| Anti-CAR Antibody | Affinity | Cat:DF6725 RRID:AB_2838687 |
| Anti- CD45.1 Antibody | Biolegend | Cat: 110702 RRID:AB_313491 |
| DAPI | Thermo Fisher Scientific | Cat:D3571 RRID:AB_2307445 |
| CoraLite594 conjugated Goat Anti-Mouse IgG(H+L) Antibody | Proteintech | Cat:SA00013-3 RRID:AB_2707133 |
| Goat Anti-Rabbit IgG (H+L) Fluor647-conjugated Antibody | Affinity | Cat:S0013 RRID:AB_2844801 |
| CoraLite594 conjugated Goat Anti-Rabbit IgG(H+L) Antibody | Proteintech | Cat:SA00013-4 RRID:AB_2810984 |
| Anti-rat IgG (H+L), (Alexa Fluor 488 Conjugate) Antibody | Cell Signaling Technology | Cat:4416 RRID:AB_10693769 |
| ELISA kits |  | |
| Mouse IL-35 | mlbio | Cat: ml063154 |
| Mouse IL-27 | R&D | Cat: M2728 |
| Mouse IL-12 | R&D | Cat: M1270 |
| Mouse IL-10 | R&D | Cat: M1000B |
| Mouse IAA | mlbio | Cat: ml401842 |
| Mouse LPS | Biocompare | Cat: ABIN6200457 |
| Human IL-35 | mlbio | Cat: ml058088 |
| Human IAA | mlbio | Cat: ml062551 |
| Reagents |  | |
| Trizol | Life technologies | Cat:15596018 |
| LPS (O111:B4) | Sigma | Cat:L2630 |
| IAA | MCE | Cat:HY-18569 |
| PXR inhibitor | MCE | Cat:HY-16561 |
| AHR inhibitor | MCE | Cat:HY-12684 |
| NF-κB inhibitor | MCE | Cat:HY-14592 |
| ProteinA/G Magnetic Beads | MCE | Cat:HY-K02 |
| Nuclear and Cytoplasmic Protein Extraction Kit | Beyotime | Cat: P0028 |
| L-Tryptophan | MCE | Cat:HY-N0623 |
| Oligonucleotides for qRT-PCR |  | |
| Murine GAPDH FW | BGI | 5’-TCAACGGCACAGTCAAGG-3’ |
| Murine GAPDH REV | BGI | 5’-TACTCAGCACCGGCCTCA-3’ |
| Murine TNFa FW | BGI | 5’-CCAGACCCTCACACTCAGATCA-3’ |
| Murine TNFa Rev | BGI | 5’-GTAGACAAGGTACAACCCATCG-3’ |
| Murine IL-6 FW | BGI | 5’-ACAACCACGGCCTTCCCTACTT-3’ |
| Murine IL-6 Rev | BGI | 5’-TTTCTCATTTCCACGATTTCCC-3’ |
| Murine MCP-1 FW | BGI | 5’- TTCTTCGATTTGGGTCTCCTTG-3’ |
| Murine MCP-1 Rev | BGI | 5’- GTGCAGCTCTTGTCGGTGAA-3’ |
| Murine Ebi3 FW | BGI | 5’- CATTGCCACTTACAGGCTCG-3’ |
| Murine Ebi3 Rev | BGI | 5’- TGCAGTGACATTTAGCATGTAGG-3’ |
| Murine p35 FW | BGI | 5’- TGCCTTGGTAGCATCTATGAGG-3’ |
| Murine p35 Rev | BGI | 5’- CGCAGAGTCTCGCCATTATGAT-3’ |
| Murine TGF-β FW | BGI | 5’- CCACCTGCAAGACCATCGAC -3’ |
| Murine TGF-β Rev | BGI | 5’- CTGGCGAGCCTTAGTTTGGAC -3’ |
| Murine IL-10 FW | BGI | 5’-AGCCTTATCGGAAATGATCCAGT -3’ |
| Murine IL-10 Rev | BGI | 5’- GGCCTTGTAGACACCTTGGT -3’ |
| Human Reg4 FW | BGI | 5’- CTGCTCCTATTGCTGAGCTG -3’ |
| Human Reg4 Rev | BGI | 5’-GGACTTGTGGTAAAACCATCCAG-3’ |
| Human GAPDH FW | BGI | 5’-GTCAAGGCTGAGAACGGGAA-3’’ |
| Human GAPDH Rev | BGI | 5’-AAATGAGCCCCAGCCTTCTC-3’ |
| Oligonucleotides for mutated lactobacillus |  | |
| iaaM-up-FW | BGI | 5’-CCGCTCGAGAACGTTTTCCATCAAGTTGAGC-3’ Xho ǀ |
| iaaM-up-RW | BGI | 5’-AGCTTTGTTTAAACAACTGACTATTCACCACGCCTC-3’ Pme ǀ |
| iaaM-down-FW | BGI | 5’-CGAGCTCAATCACACACAATCAACTATGGACA-3’ Sac ǀ |
| iaaM-down-RW | BGI | 5’-GAAGATCTGCAAGTCCCGTTTGAACATCT-3’ Bgl Ⅱ |
| iaaM-FW | BGI | 5’-ATGACCAAAACCAACTATATCAATG-3’ |
| iaaM-RW | BGI | 5’-TTTAGCCGCTTGATGAACTTGA-3’ |
| Cre-FW | BGI | 5’-CTAACTCGAGTGATCACCAATTC-3’ |
| Cre-RW | BGI | 5’-GGCTATCAATCAAAGCAACACG-3’ |
| CM-FW | BGI | 5’-ATGAACTTTAATAAAATTGATTTAGACAATTG-3’ |
| CM-RW | BGI | 5’-TTATAAAAGCCAGTCATTAGGCCTATC-3’ |
| Oligonucleotides for CHIP-PCR |  | |
| PXR-P35-FW | BGI | 5’-TAGGGACTGTGTCTGGTGGC-3’ |
| PXR-P35-RW | BGI | 5’-ATTTGGAGGTTTGGGGTAGG-3’ |
| Bacteria primers |  |  |
| 16s 27F | BGI | 5’- AGAGTTTGATCCTGGCTCAG-3’ |
| 16s 1492R | BGI | 5’- GGTTACCTTGTTACGACTT-3’ |
| Eubacteria-Fs | BGI | 5’- ACTCCTACGGGAGGCAGCAGT-3’ |
| Eubacteria-Rs | BGI | 5’-ATTACCGCGGCTGCTGGC-3’ |
| Firmicutes-Fs | BGI | 5’-GCTGCTAATACCGCATGATATGTC-3’ |
| Firmicutes-Rs | BGI | 5’-CAGACGCGAGTCCATCTCAGA-3’ |
| Bacteroidetes-Fs | BGI | 5’-GAGAGGAAGGTCCCCCAC-3’ |
| Bacteroidetes-Rs | BGI | 5’-CGCTACTTGGCTGGTTCAG-3’ |
| Total Lactobacillus-Fs | BGI | 5’-AGCAGTAGGGAATCTTCCA -3’ |
| Total Lactobacillus-Rs | BGI | 5’-CACCGCTACACATGGAG -3’ |
| L.Reuteri-Fs | BGI | 5’-ACCGAGAACACCGCGTTATTT -3’ |
| L.Reuteri-Rs | BGI | 5’-CATAACTTAACCTAAACAATCAAAGATTGTCT -3’ |
| L. acidophilus-Fs | BGI | 5’-GAAAGAGCCCAAACCAAGTGATT -3’ |
| L. acidophilus-Rs | BGI | 5’-CTTCCCAGATAATTCAACTATCGCTTA -3’ |
| L.Murinus-Fs | BGI | 5’-AGCTAGTTGGTGGGGTAAAG -3’ |
| L.Murinus-Rs | BGI | 5’-TAGGATTGTCAAAAGATGTC -3’ |
| iaaM-Fs | BGI | 5’-GGGGTAAAAGAGGCGGTTCA -3’ |
| iaaM-Rs | BGI | 5’-GATTACCTTTTCACGCGCCC-3’ |
| iaaH-FW | BGI | 5’-GAAATCAGCTTCGTGCTGGC-3’ |
| iaaH-RW | BGI | 5’ GTCGCGATAGATGCCGATCA-3’ |
| Oligonucleotides for IL-35 siRNAs |  | |
| mIL-12A -702 | Sense | 5’-GCAGACCCUUACAGAGUGAAATT-3’ |
|  | Antisense | 5’-UUUCACUCUGUAAGGGUCUGCTT-3’ |
| mIL-12A -342 | Sense | 5’-GCUGAAGACAUCGAUCAUGAATT-3’ |
|  | Antisense | 5’-UUCAUGAUCGAUGUCUUCAGCTT-3’ |
| mIL-12A -576 | Sense | 5’-GCACUUCAGAAUCACAACCAUTT-3’ |
|  | Antisense | 5’-AUGGUUGUGAUUCUGAAGUGCTT-3’ |
| Bacteria strain |  |  |
| *Lactobacillus reuteri* | BioGaaia, Sweden |  |
| *Lactobacillus* *Johnsoni* | BNCC | BNCC135265 |
| *Lactobacillus acidophilus* | BNCC |  |
| *Lactobacillus animalis* | BNCC |  |
| Other |  |  |

| Other reagents | | |
| --- | --- | --- |
| Ampicillin | Sigma | Cat: BP021 |
| Vancomycine | Sigma | Cat: V2002 |
| Gentamicin | Sigma | Cat: E003632 |
| Neomycin sulfate | Sigma | Cat: N6386 |
| Metronidazole | Sigma | Cat: M3761 |
| Eflornithine (DFMO) | MCE | Cat: HY-B0744B |
| High-fat diet | Research Diets | Cat: D12492 |
| Rogosa SL selective medium | Sigma | Cat: R1148 |
| MRS | 3M US | Cat: BP0275500 |
| Trizol | Life Technologies | Cat: 15596026 |
| QIAquick PCR Purification Kit | Qiagen | Cat:28104 |
| QuantiTect SYBR Green PCR Master Mix | Qiagen | Cat:208052 |
| FBS | Gibco | Cat:10099141 |
| Collagenase IV | Sigma | Cat: C5138 |
| Collagenase I | Sigma | Cat: SCR103 |
| Dnase I | Solarbio | Cat: D8071 |
| DMEM | Gibco | Cat:11965118 |
| HBSS | Gibco | Cat:14170161 |
| FICOLL | Sigma | Cat: 341691 |
| Percoll | Sigma | Cat: P4937 |
| Cell stimulation cocktail | eBioscience | Cat: 00-4975-03 |
| Foxp3 fix/perm buffer | Biolegend | Cat: 421403 |
| PMA | Sigma | Cat: 79346 |
| GolgiStop | BD Biosciences | Cat: 554724 |
| Permeabilization Buffer | eBioscience | Cat: 00-8333-56 |
